# Modeling hepatitis A epidemiological profiles and estimating the pediatric vaccination threshold in the Russian Federation

**DOI:** 10.3389/fpubh.2024.1371996

**Published:** 2024-06-27

**Authors:** Fedor F. Taratorkin, Anastasia A. Karlsen, Karen K. Kyuregyan, Maria A. Lopatukhina, Farhad Khankishiyev, Victor A. Manuylov, Vasiliy G. Akimkin, Mikhail I. Mikhailov

**Affiliations:** ^1^Mechnikov Research Institute of Vaccines and Sera, Moscow, Russia; ^2^Central Research Institute of Epidemiology, Moscow, Russia; ^3^Faculty of Public Health, Organization and Sociology of Healthcare and Medical-Social Expertise, Moscow, Russia; ^4^Gamaleya National Research Center for Epidemiology and Microbiology, Moscow, Russia

**Keywords:** hepatitis A, HAV, herd immunity, hepatitis A vaccination, mathematical model

## Abstract

**Background:**

To combat the hesitancy towards implementing a hepatitis A universal mass vaccination (UMV) strategy and to provide healthcare authorities with a comprehensive analysis of the potential outcomes and benefits of the implementation of such a vaccination program, we projected HAV seroprevalence and incidence rates in the total population of the Russian Federation and estimated the pediatric vaccination threshold required to achieve an incidence level of less than 1 case per 100,000 using a new mathematical model.

**Methods:**

A dynamic age-structured SEIRV (susceptible-exposed-infectious-recovered-vaccinated) compartmental model was developed and calibrated using demographic, seroprevalence, vaccination, and epidemiological data from different regions of the Russian Federation. This model was used to project various epidemiological measures.

**Results:**

The projected national average age at the midpoint of population immunity increases from 40 years old in 2020 to 50 years old in 2036 and is shifted even further to the age of 70 years in some regions of the country. An increase of varying magnitude in the incidence of symptomatic HAV infections is predicted for all study regions and for the Russian Federation as a whole between 2028 and 2032, if the HAV vaccination coverage level remains at the level of 2022. The national average vaccination coverage level required to achieve a symptomatic HAV incidence rate below 1 case per 100,000 by 2032 was calculated to be 69.8% if children aged 1–6 years are vaccinated following the implementation of a UMV program or 34.8% if immunization is expanded to children aged 1–17 years.

**Conclusion:**

The developed model provides insights into a further decline of herd immunity to HAV against the background of ongoing viral transmission. The current favorable situation regarding hepatitis A morbidity is projected to be replaced by an increase in incidence rates if vaccination coverage remains at the current levels. The obtained results support the introduction of a hepatitis A UMV strategy in the Russian Federation.

## 1 Introduction

Hepatitis A is a self-limited acute liver disease that is caused by a single-stranded RNA virus; the Hepatitis A virus (HAV, also known as Hepatovirus A) is a member of the *Picornaviridae* family. Five HAV genotypes are recognized, of which three genotypes are divided into subtypes A and B (HAV-IA, IB, IIA, IIB, IIIA, and IIIB) and infect humans, while genotypes IV and V cause infection in monkeys (1). Hepatitis A is a ubiquitous infection and occurs in the form of both sporadic cases and outbreaks. Globally, approximately 1.5 million cases of symptomatic acute hepatitis A are reported each year, resulting in 15,000 to 30,000 deaths per year (2). The clinical manifestations of hepatitis A largely depend on the age of the infected individual and the presence of comorbidities. Typically, HAV infection in children under 5 years is asymptomatic, while in adults, hepatitis A presents moderate-to-severe clinical manifestations (3). Patients with comorbid liver disease, including chronic viral hepatitis, or immunosuppressed individuals have an increased risk of HAV-associated liver failure and fatal outcome (4). HAV is typically acquired through fecal-oral transmission, which is associated with the viral contamination of food or drinking water, as well as via direct person-to-person contacts (5). Hepatitis A endemicity is classified based on the prevalence of IgG antibodies to HAV (anti-HAV) in a general population, which is categorized as high (≥90% by the age of 10 years), intermediate (≥50% by the age of 15 years and <90% by the age of 10 years), low (≥50% by the age of 30 years and <50% by the age of 15 years), and very low (<50% by the age of 30 years) (2). Changes in hepatitis A epidemiology that occur in many countries are associated with an improvement in sanitation and the availability of high-quality drinking water, which lead to a decrease in the circulation of HAV among children and, as a result, an increase in the proportion of susceptible adolescents and young adults. Such a transition from high hepatitis A endemicity to intermediate endemicity causes an increase in the number of non-immune adults and, thus, an increase in the number of clinically significant cases of hepatitis A and associated hospitalizations against the background of a general decrease in the reported incidence (6, 7). This situation is highly typical of Russia, where over the past decade, a significant drop in the registered incidence of hepatitis A to a rate below 10 cases per 100,000 per year has been observed. As a result, a consistent decrease in herd immunity to HAV has occurred, especially among adolescents and adults under 30 years old, in whom anti-HAV antibody prevalence is less than 50% (8).

Hepatitis A is a vaccine-preventable disease. According to the WHO recommendations, the indications for the introduction of an HAV universal mass vaccination (UMV) strategy into national immunization schedules are as follows: (i) an increasing trend over time of acute hepatitis A disease, including severe disease, among older children, adolescents, or adults; (ii) changes in endemicity from high to intermediate; and (iii) considerations of cost-effectiveness (9). The experience of countries that have introduced a universal pediatric HAV vaccination program indicates that this approach is highly effective in rapidly reducing the burden of this disease (10).

In Russia, vaccination against hepatitis A is carried out for risk groups and epidemically significant populations (healthcare workers, sewerage workers, staff in institutions providing care for people with mental or behavioral disorders, childcare facility staff, and workers in food service), as well as for contact personnel in the management of outbreaks, since post-exposure vaccination within 2 weeks can prevent the disease (11). Several inactivated HAV vaccines are registered in Russia, both from domestic and international manufacturers. A UMV strategy has not been implemented at the national level so far. However, there are regional vaccination programs in several parts of the country (Sverdlovsk Region, Yakutia, and Tuva), but their contribution to changes in herd immunity to HAV has been shown to be insignificant on a national scale (8). The hesitancy towards a hepatitis A universal vaccination program among healthcare policymakers is mainly due to the decrease in HAV incidence rates over the last decade and a widely adopted opinion that hepatitis A is not a significant disease, given its low morbidity and mortality rates.

Several studies addressed the shift in HAV endemicity using mathematical models to predict the changes in HAV seroprevalence and incidence over time (12, 13). These studies, based on regional HAV seroprevalence and demographic data, demonstrated no projected reduction in symptomatic cases in the absence of vaccination, despite a continued decrease in the number of HAV infections (13). Moreover, epidemic modeling was shown to be a useful tool for estimating the vaccination coverage threshold required to achieve herd immunity, including in vulnerable populations (14). To combat the hesitancy towards a UMV strategy and to provide healthcare authorities with a comprehensive analysis of the potential outcomes and benefits of the implementation of such a vaccination program, we projected HAV seroprevalence and incidence rates in the Russian Federation, and estimated the vaccination threshold required to achieve an incidence level of less than 1 case per 100,000 using a new mathematical model that was applied to data from different parts of the country.

## 2 Materials and methods

### 2.1 Study design

The modeling of HAV epidemic patterns for total population was performed using a dynamic age-structured SEIRV (susceptible-exposed-infectious-recovered-vaccinated) compartmental model for eight regions spanning the territory of the Russian Federation from west to east and representing six out of the eight Federal Districts of the country, as shown in Table 1. All three Russian regions that have a regional pediatric HAV vaccination program were included in this study.

**Table 1:** Study regions and HAV vaccination strategy in each region.

Study region	Federal district of the Russian Federation	Population density (people per km^2^)	Urban population, %	HAV vaccination strategy in the study region
Saint Petersburg	Northwestern	3,991.48	100	Risk groups since 2001
Moscow	Central	5,116.82	98.4	Risk groups since 2001; children aged 3 to 6 years since 2014
Republic of Dagestan	North Caucasian	63.85	44.9	Risk groups since 2001
Sverdlovsk Region	Ural	21.82	86.3	Risk groups since 2001; children aged 6 years since 2003, children aged 20 months since 2008
Novosibirsk Region	Siberian	15.72	79.4	Risk groups since 2001
Tuva Republic	Siberian	2.00	54.7	Risk groups since 2001; children aged 3 years and older (single-dose immunization schedule) since 2012
Sakha Republic (Yakutia)	Far Eastern	0.32	67.0	Risk groups since 2001; children aged 20 months since 2011
Khabarovsk Region	Far Eastern	1.63	84.0	Risk groups since 2001

Regional-specific data on HAV seroprevalence in the general population, hepatitis A incidence, vaccination coverage in children and adults, and demographic data, as described in detail in the following subsections, were used to model the changes in HAV seroprevalence and symptomatic hepatitis A incidence for total population up to the year 2036. Modeling was also performed for the Russian Federation as a whole, using national average data on incidence, vaccination coverage, and demographics combined with data on HAV seroprevalence from the study regions. For all study regions and the Russian Federation as a whole, projections up to the year 2036 were performed using several alternative scenarios: (i) current vaccination coverage; (ii) a decrease in vaccination coverage rates by 4% per year (based on the 4% annual decrease observed over the last 10 years); and (iii) an increase in vaccination coverage rates by 4% per year. Next, based on the predicted HAV seroprevalence and incidence rates, we calculated the threshold vaccination coverage rates required to achieve an incidence rate in symptomatic infections of less than 1 case per 100,000. These projections were performed for two scenarios—when vaccination is mainly given to children aged 1–6 years, and when the vaccination coverage rates are similar among children of all age groups under 18 years.

### 2.2 HAV seroprevalence data

Data on age-specific anti-HAV IgG antibody detection rates in five study regions (Moscow, the Sverdlovsk Region, the Tuva Republic, the Sakha Republic (Yakutia), and the Khabarovsk Region) were obtained from a previous study (8). This dataset included two time points, 2008 and 2020, for each region, and additional archival data were obtained from serosurveys conducted in the Moscow Region in 1981 and 1993 using *in house* quantitative ELISA assay, which was validated against anti-HAV antibody international standard. To broaden the geographic representation of the study regions, an additional cross-sectional serosurvey was conducted using the same methodology in three regions: Saint Petersburg, the Republic of Dagestan, and Novosibirsk Region. Age-specific anti-HAV IgG antibody testing was performed in sera collected in 2020 from healthy volunteers in these three regions and separated into the following age groups: 1–9 years, 10–14 years, 15–19 years, 20–29 years, 30–39 years, 40–49 years, 50–59 years, and 60+ years. The total number of study participants was 5,328 in Saint Petersburg, 4,859 in the Republic of Dagestan, and 8,331 in the Novosibirsk Region. The sample size was calculated with the chosen power (80%) and confidence level (95%) based on the known size of the population in these regions and data on anti-HAV antibody prevalence rates in other regions of Russia. The mean sample size in each age group was 772 individuals (113–1961), with the mean male/female ratio being 1:1.1 and varying between 1:0.9 and 1:1.6 depending on the age group.

The healthy volunteers were persons undergoing routine medical examinations, visitors to a vaccination center undergoing routine vaccinations, and patients visiting a polyclinic for reasons not related to infectious diseases. The inclusion criteria were permanent residence in one of the study regions and provision of a signed and dated informed consent form approved by the Ethics Committee. The following exclusion criteria were applied: children in care, treatment with blood products within 3 months before registering for the study (self-reported or parent-reported), and a body temperature over 37.10°C or an acute illness. This study was conducted in accordance with the principles laid out in the World Medical Association’s Declaration of Helsinki regarding ethical medical research involving human subjects. Written informed consent was obtained from all participants or their parents (or legal guardians). The study design was approved by the Independent Interdisciplinary Ethics Committee for the Ethical Review of Clinical Research, Moscow, Russia (Approval No. 17 dated November 16, 2019).

All sera from the healthy volunteers were tested for anti-HAV IgG antibodies using the same commercially available ELISA Vecto-hep А-IgG kit (Vector-Best, Novosibirsk, Russia) that was used in the previous serosurveys (8). Testing was performed according to the kit manufacturer’s instruction. Samples with anti-HAV concentrations ≥20 mIU/mL were considered positive.

The 95% confidence intervals (95% CI) for the proportions of seropositive individuals were calculated GraphPad 10.0.2 software.^1^

### 2.3 HAV incidence and vaccination coverage data

National average and region-specific data on the annual incidence of hepatitis A from 1999 to 2022 were retrieved from the annual federal statistical forms 1 on infectious and parasitic disease morbidity, which were issued by the Russian Federal Service for Surveillance on Consumer Rights Protection and Human Wellbeing (Rospotrebnadzor). These data are presented as cases per 100,000 for the total population and separately for children aged 0–14 years, children aged 0–17 years, and adults (18 years and older) in Supplementary Table S1.

National average and region-specific data on annual HAV vaccination rates from 2004 to 2022 were retrieved from the federal statistical forms 5 on vaccination against infectious diseases, which were issued annually by Rospotrebnadzor. These data are presented in Supplementary Table S2 as absolute numbers of vaccinations and as numbers per 100,000 for the total population and separately for children aged 0–17 years and adults as well.

### 2.4 Demographic data

As the proportion of HAV symptomatic cases varies significantly by age and the proportion of persons who acquire life-long immunity increases with age, the age-structured model is heavily dependent on the input demographic data. For HAV epidemic modeling, the population of each study region was split into 1 year age groups, from 0 to 85+ years.

Data from the Federal State Statistic Service (Rosstat) (15) were used to build a demographic model. However, the demographic projections presented by Rosstat for several study regions were given only for broader age groups or for the total population, while the age-structured model required the size of each one-year age group. Thus, we built our own predictive demographic model using the following input data from the Rosstat website for each study region: the available data on the size of each one-year age group as of January 1, 2021; the predicted number of newborns from 2021 to 2036; mortality by age groups from 2008 to 2019; and the number of immigrants and emigrants in 2017, 2018, 2019, and 2022. Next, using linear extrapolation, expected mortality rates by age groups from 2021 to 2036 were calculated. The age distributions of mortality for each year were calculated based on an extrapolation of mortality per 100,000 for 5 year age groups in the period from 2008 to 2019, with the year 2020 being excluded from the calculation due to COVID-19-related changes in mortality. The number of deaths in each age group was calculated by applying its distribution to the number of deaths expected by Rosstat. The number of immigrants and emigrants was taken as an average for the years 2017–2019. The size of each one-year age group in each subsequent year, starting from 2022, was calculated as the size of the previous age group in the current year while subtracting mortality and taking into account migration (a diagonal shift).

The following equation was used to estimate the size of each age group:


$$N_{a+1,i+1}=N_{a,i}*\left(1+I_{a}-E_{a}-M_{a,i}\right)$$

where $N_{a,i}$ is the number of age group *a* in year *i*, $I_{a}$ is the relative number of immigrants of age *a*, $E_{a}$ is the relative number of emigrants of age *a*, and $M_{a,i}$ is the relative number of deaths in age group *a* in year *i*.

All variables from Rosstat used in calculations contained 95% confidence interval.

As a result, a table containing values indicating the size of each 1 year age group from 2021 to 2036 was obtained. To check the validity of our projections, the total population at the beginning of each year was calculated and graphs of the total population were constructed based on our own model and the Rosstat projected data.

### 2.5 Prediction of age-specific HAV seroprevalence rates

Age-specific HAV seroprevalence curves for all age groups of the population for 2008 and 2020 were obtained by fitting the data described in subsection 2.2. Several variants of approximated graphs were tested (Gompertzian, logistic, hyperbolic tangent and some other trigonometric functions, 4th and more degree polynomials). The function with the minimal value of the highest minimal sum of squares was selected as optimal. This option was chosen to obtain maximum convergence between regions. For example, the logistic sigmoid gave the best approximation in most regions, but in the Sverdlovsk Region it was very far from the original curve. The best average covariance parameters for all regions were shown by a 4th-degree polynomial: a*×^4 + b*×^3 + c*×^2 + d*× + e. Based on the obtained functions, the absolute number of persons having anti-HAV IgG antibodies in each age group was calculated. These values were then modified by the SEIRV model based on the transition rates between compartments and used to generate expected seroprevalence plots.

### 2.6 SEIRV model

The modeling of epidemiological dynamics was performed using an age-structured SEIRV compartmental model, a modification of the SIR (Susceptible-Infected-Recovered model by Kermack andMcKendrick) model with 430 groups for each study region and for Russia as a whole. This model was chosen as it shows good accuracy when there is a small number of symptomatic cases for a disease at the starting point, as was reported for HAV over the past years. The population of each region was split into 86 age groups and 5 compartments.

The dynamics of each compartment follows the system of differential equations:


$$\frac{dS_{a}}{dt}=-\beta \sum I_{a}*\frac{S_{a}}{N_{a,i}}-dV_{a}*\left(1-\frac{R_{a}}{N_{a,i}}\right)+\frac{S_{a-1}N_{a,i+1}dt}{N_{a-1,i}Y}-\frac{S_{a}dt}{Y}$$

$$\frac{dE_{a}}{dt}=\beta \sum I_{a}*\frac{S_{a}}{N_{a,i}}-\alpha E_{a}$$

$$\frac{dI_{a}}{dt}=\alpha E_{a}-\gamma I_{a}$$

$$\frac{dR_{a}}{dt}=\gamma I_{a}-dV_{a}*\frac{R_{a}}{N_{a,i}}+\frac{R_{a-1}N_{a1,i+1}dt}{N_{a-1,i}Y}-\frac{R_{a}dt}{Y}$$

$$\frac{dV_{a}}{dt}=\varepsilon v_{t}+\frac{V_{a-1}N_{a1,i+1}dt}{N_{a-1,i}Y}-\frac{V_{a}dt}{Y}$$

where:

*S*—Susceptible people who are not infected nor vaccinated, are unable to transmit infection, but are able to be infected or vaccinated. This compartment is replenished by newborns or susceptible people from younger age groups.

*E*—Exposed people who are infected, but still not infectious, and who pass to the infectious group after a fixed period of time. This parameter is defined as the time between being infected and the start of viral shedding, which is usually about 10 days (16).

*I*—Infectious and diseased cases, including asymptomatic cases, who are able to infect others.

*R*—Recovered people who are unsusceptible to the virus and are unable to infect or be infected due to life-long immunity from past infection.

*V*—Vaccinated people who received immunity through vaccination and are considered to have life-long immunity.

The number of people in the *R* and *V* groups decrease only because of aging or death.

In the first equation, which shows changes in the number of susceptible people of age *a* $,\beta \sum I_{a}*\frac{S_{a}}{N_{a,i}}$ is the number of new infections, where β is the average number of contacts with one infectious person in a period of illness; $\Sigma I_{a}$ is the total number of infectious people at the current moment for all age groups; $S_{a}$ is the number of susceptible people of age *a*; and $N_{a,i}$ is the number of people in age group *a* in year *i*.

$dV_{a}*\left(1-\frac{R_{a}}{N_{a,i}}\right)$ is the number of vaccinated individuals in a period of time, considering that recovered persons can be vaccinated as well.

$\frac{S_{a-1}N_{a1,i+1}dt}{N_{a-1,i}Y}-\frac{S_{a}dt}{Y}$ is the change related to aging, where $S_{a-1}$ and $N_{a-1,i}$ are the numbers of susceptible people and all people 1 year younger than this age group.

$N_{a,i+1}$ is the number of people in an age group in the next year, and *Y* denotes 1 year.

In the second equation, $\frac{dE_{a}}{dt}\;$ indicates the change in the exposed compartment.

$\beta \sum I_{a}*\frac{S_{a}}{N_{a,i}}$ is the number of new cases.

$\alpha E_{a}$ is a function of passing from being exposed to being infectious.

The third equation shows the dynamics of the infectious group, where $\alpha E_{a}$ is the number of people passed from the exposed compartment and $\gamma I_{a}$ is the number of people passed to being recovered.

In the fourth equation, $\gamma I_{a}$ is the number of recently recovered people from infection.

$dV_{a}*\frac{R_{a}}{N_{a,i}}$ shows the number of recovered individuals who received vaccine, and $\frac{R_{a-1}N_{a1,i+1}dt}{N_{a-1,i}Y}-\frac{R_{a}dt}{Y}$ represents aging as in the first equation.

The last equation shows changes in the number of vaccinated individuals in each age group based on a function of the distribution of the number of doses $\varepsilon v_{t}$ and aging of vaccinated individuals, $\frac{V_{a-1}N_{a1,i+1}dt}{N_{a-1,i}Y}-\frac{V_{a}dt}{Y}$ .

### 2.7 Pediatric HAV vaccination scenarios

The above equation system was first run with the floating parameter β and the numbers of compartments to obtain projections close to the real incidence rate dynamics observed in 2008–2020, i.e., the period of time when HAV seroprevalence data were available. Next, using the obtained parameters, calculations were performed to predict the dynamics of hepatitis A incidence in total population up to the year 2036 under three different vaccination scenarios: vaccination rates as in the year 2022; an increase in vaccination rates by 4% per year; and a decline in vaccination rates by 4% per year (tendency observed in the country within last decade).

Next, using the same equation system, two scenarios of pediatric vaccination required to achieve the target incidence rate of less than 1 case per 100,000 persons per year were investigated. In the first scenario, vaccination was carried out mainly among children aged 1–6 years. In the second scenario, vaccination coverage was even among children aged 1 to 17 years.

To calculate the required vaccination coverage rates in both scenarios, the variable responsible for the increase in annual vaccination rates had an increment of 100 full vaccinations per program run. The program was run repeatedly until the final projected incidence rate fell below 1 case per 100,000 after 2032.

## 3 Results

### 3.1 Demographic model

**Figure 1:**
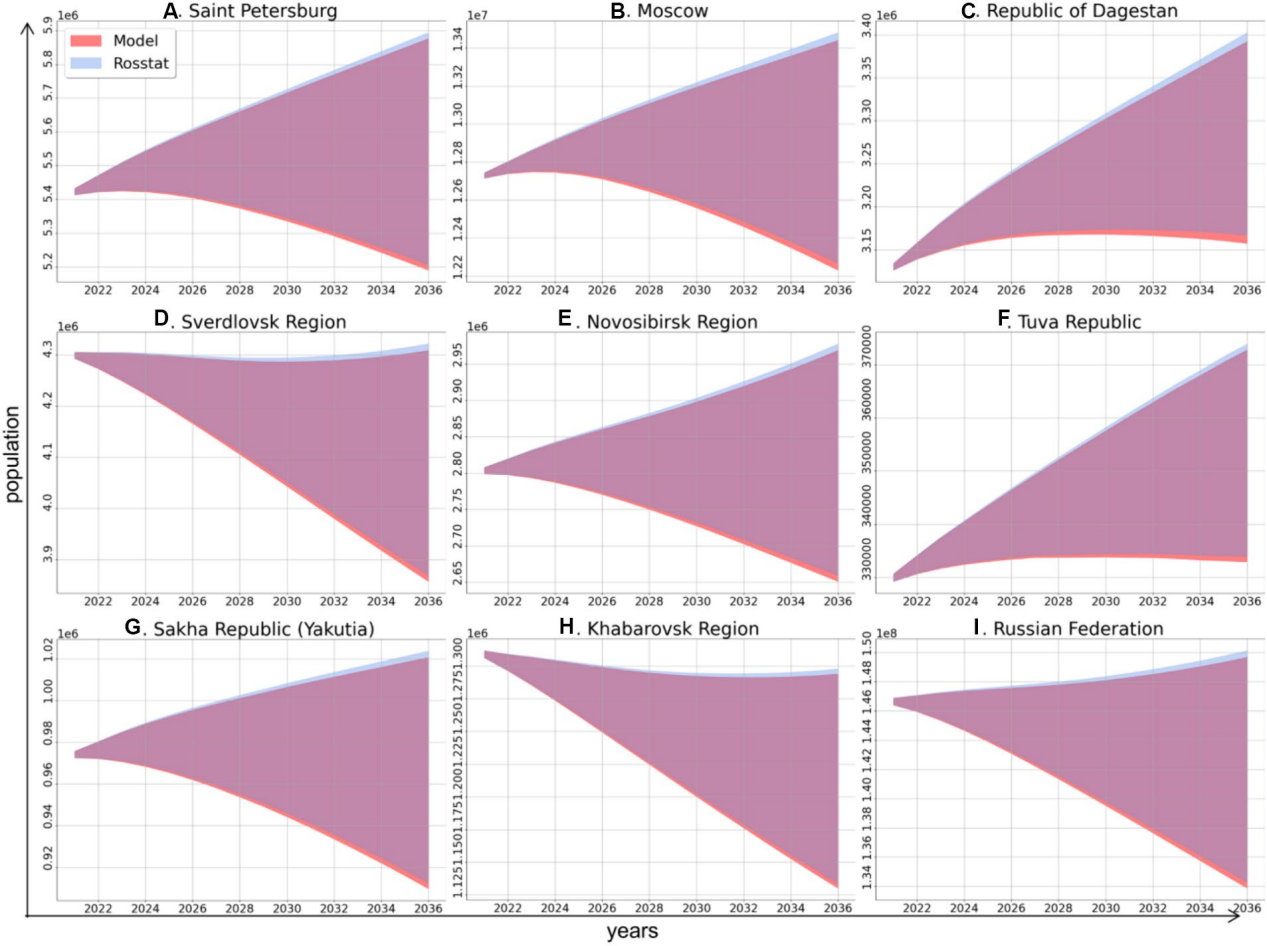
Comparison of the dynamics of total population size in the study regions and in the Russian Federation as a whole in 2021–2036 according to the model of this study (red area) and based on data from Rosstat (blue area), these projections have high similarity (overlapping areas are shown in purple).

The first stage of simulation was running the demographic model for each of the study regions and for the Russian Federation as a whole up to the year 2036. This modeling was performed to obtain the value of the population size for each age group, which was estimated using a unified method. Next, the predicted values were compared to the dynamics of the total population predicted by Rosstat to validate our demographic model. For each study region and for the country as a whole, the demographic model shows 99.17% agreement with the Rosstat projections on population size (Figure 1).

The next stage of modeling was to obtain the model parameters from existing data for the years 2008–2020, i.e., the period of time when HAV seroprevalence data were collected. The obtained values for the coefficient β (average number of people contacting one infectious person throughout the course of infection) are shown in Table 2. This coefficient was on average about 2 for the Russian Federation and varied from 1.51 to 3.52 between regions. All study regions could be stratified based the values of coefficient β : the values were around 1.5 for megacities such as Moscow and Saint Petersburg, around 2 for urbanized regions such as Sverdlovsk Region and Khabarovsk Region, and 3 or higher for rural regions.

**Table 2:** Values of coefficient β in the study regions and in the Russian Federation as a whole.

Study region	Coefficient β value
Saint Petersburg	1.55
Moscow	1.51
Republic of Dagestan	3.00
Sverdlovsk Region	2.36
Novosibirsk Region	1.64
Tuva Republic	3.52
Sakha Republic (Yakutia)	3.25
Khabarovsk Region	1.95
Russian Federation, average	2.03

### 3.2 Projected HAV age-specific seroprevalence rates

The age-specific anti-HAV IgG detection rates in healthy volunteers from the three regions (Saint Petersburg, the Republic of Dagestan, and the Novosibirsk Region) surveyed in this study are shown in Table 3. The anti-HAV prevalence data used for modeling other study regions were retrieved from previous serosurveys (8).

**Table 3:** Age-specific anti-HAV IgG detection rates in healthy volunteers from the three study regions.

Study region		Age group, years								Total
		1–9	10–14	15–19	20–29	30–39	40–49	50–59	≥60	
Saint Petersburg	Number of persons	354	113	129	1,455	1961	669	401	246	5,328
	anti-HAV IgG(+), *n*	43	14	20	226	454	232	207	189	1,385
	% seropositive	12.1%	12.4%	15.5%	15.5%	23.2%	34.7%	51.6%	76.8%	26.0%
	95% CI, ±	3.4%	6.1%	6.2%	1.9%	1.9%	3.6%	4.9%	5.3%	1.2%
Republic of Dagestan	Number of persons	1,256	639	545	755	550	277	307	530	4,859
	anti-HAV IgG(+), *n*	284	202	203	440	493	266	296	517	2,701
	% seropositive	22.6%	31.6%	37.2%	58.3%	89.6%	96.0%	96.4%	97.5%	55.6%
	95% CI, ±	2.3%	3.6%	4.1%	3.5%	2.5%	2.3%	2.1%	1.3%	1.4%
Novosibirsk Region	Number of persons	1,398	698	913	1,602	1,615	829	519	757	8,331
	anti-HAV IgG(+), *n*	104	59	100	204	440	385	299	684	2,275
	% seropositive	7.4%	8.5%	11.0%	12.7%	27.2%	46.4%	57.6%	90.4%	27.3%
	95% CI, ±	1.4%	2.1%	2.0%	1.6%	2.2%	3.4%	4.3%	2.1%	1.0%

**Figure 2:**
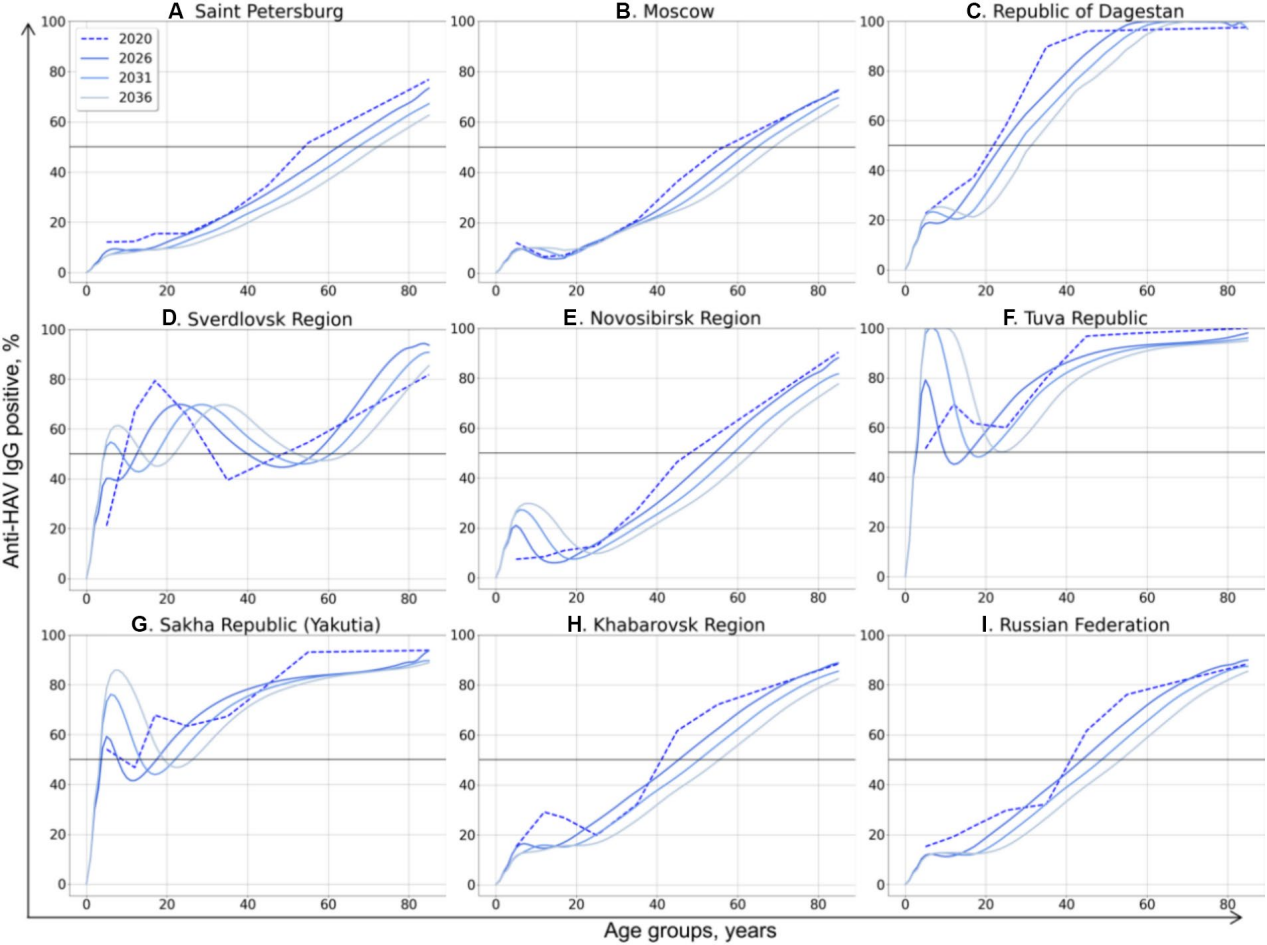
Model-projected age-specific HAV prevalence in the study regions of the Russian Federation **(A–H)** and in the country as a whole **(I)**. Current HAV seroprevalence based on data from the 2020 serosurvey is shown as dotted curves. Model-projected HAV seroprevalence data are shown as solid lines in dark blue for 2026, medium blue for 2031, and light blue for 2036. The 50% HAV seroprevalence level is shown as a black solid line.

The model-projected dynamics of age-specific anti-HAV antibody prevalence in all age groups is shown in Figure 2, together with data on current seroprevalence obtained from the 2020 serosurvey.

The HAV seroprevalence curves vary between different regions, with two types of curves distinguishable: (i) a sigmoidal or linear curve in regions where no regional HAV vaccination program has been implemented so far and in the Russian Federation as a whole, and (ii) more complex patterns in three regions where a pediatric HAV vaccination program exists (the Tuva Republic, the Sakha Republic (Yakutia), and the Sverdlovsk region).

**Figure 3:**
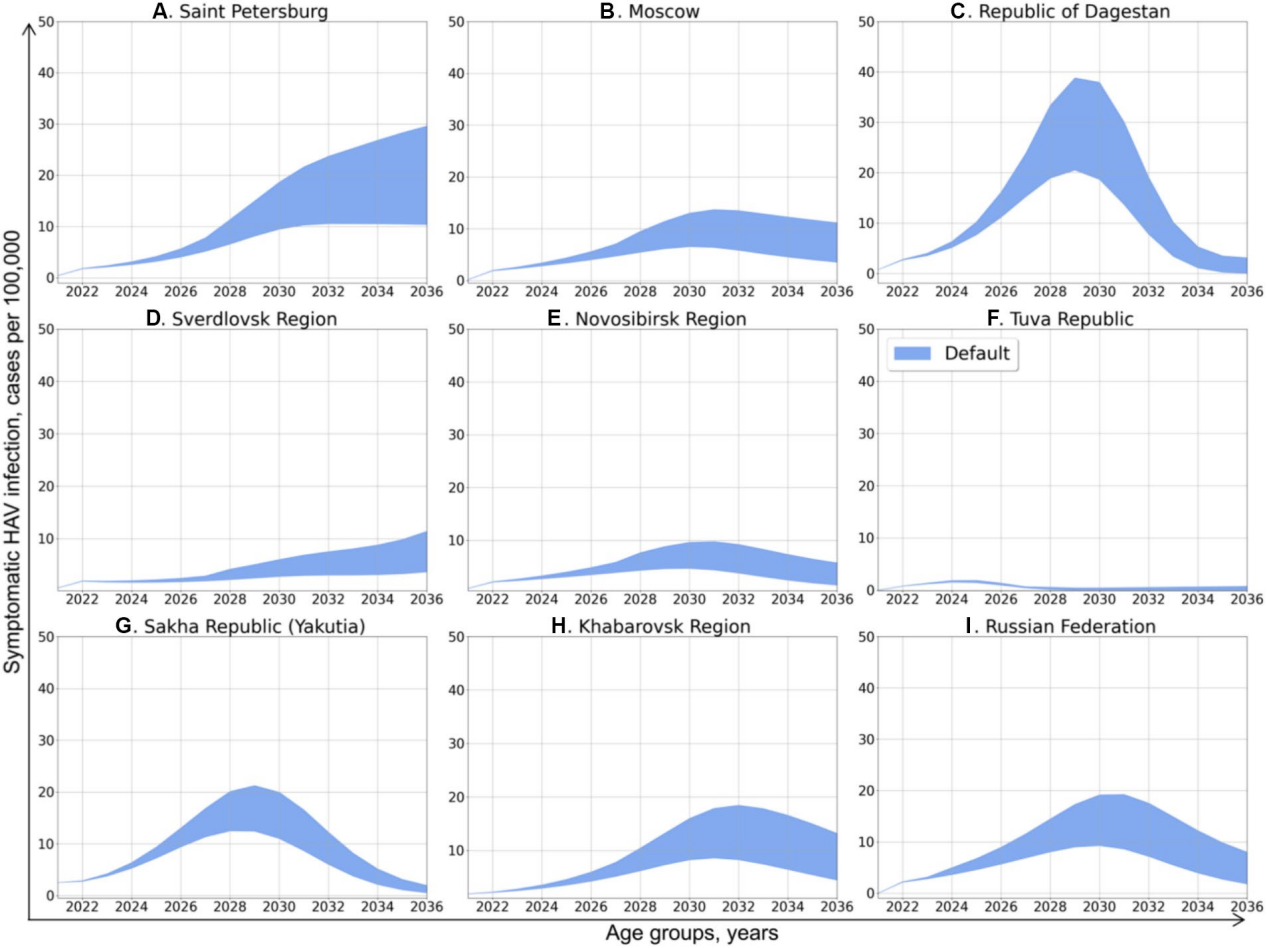
Projected dynamics of symptomatic HAV infection incidence rates in the study regions and in the Russian Federation as a whole. The blue areas depict the number of cases per 100,000 persons per year, with inaccuracy related to approximation and demographic models confidence interval.

In general, the projected HAV seroprevalence rates decline over time in all adult age groups in regions where child vaccination is not implemented, including Saint Petersburg (Figure 2A), Moscow (Figure 2B), the Republic of Dagestan (Figure 2C), the Novosibirsk Region (Figure 2E), and the Khabarovsk Region (Figure 2H). The projected HAV seroprevalence rates in children in these regions remain stable up to the year 2036, except for the Novosibirsk Region, where a gradual increase in HAV seroprevalence over time is projected (Figure 2E). The same projection is obtained for the Russian Federation as a whole, with stable and low HAV seroprevalence in children and a gradual decrease in adult age groups (Figure 2I). Because of this tendency, the first age with at least 50% HAV-positive rate (AMPI) is shifted gradually to older age groups over time. The projected national average AMPI increases from 40 years old in 2020 to 50 years old in 2036 (Figure 3I) and is shifted even further to the age of 70 years in Saint Petersburg (Figure 2A), Moscow (Figure 2B), and the Novosibirsk Region (Figure 2E).

The Sverdlovsk Region (Figure 2D) has the most complex graph, apparently due to the regional HAV vaccination program that started in 2003 for children aged 6 years and had switched to children aged 20 months since 2008. The current 70% seroprevalence rates observed in this region in adolescents are projected to shift gradually to older age groups. This projected shift is accompanied with a projected drop in HAV seroprevalence rates in adolescents in 2026–2036, probably due to the decrease in vaccination coverage observed in the region within the past years. However, the projected HAV seropositivity rates in the Sverdlovsk Region remain above 40% for the entire projection period.

In the Tuva Republic (Figure 2F), HAV seroprevalence rates in adults over 25 years decline over time in a fashion similar to that observed in other study regions. In children, however, HAV seroprevalence is projected to rise from the current rate of 80 to 100% because of vaccination. This seroprevalence peak in children, together with the high levels of anti-HAV detection rates in adult age groups, maintains the herd immunity threshold at 50% over the projection period.

In Yakutia (Figure 2G), the curves representing current and projected HAV seroprevalence are similar to those obtained for Tuva. However, the rate of HAV seroprevalence in children is lower in Yakutia, with the 85% seropositivity level being reached only by 2036.

### 3.3 Projected HAV incidence rates

After obtaining the values of coefficient β and predicting the changes in herd immunity to HAV, we performed the projection of symptomatic HAV incidence rates in total population under the first scenario, in which the rate of vaccination was assumed to remain equal to the year 2022. The predicted incidence dynamics is shown in Figure 3. An increase of various magnitude in the incidence of symptomatic HAV infection is predicted for all study regions and for the Russian Federation as a whole. Depending on the study region, these incidence graphs exhibit a shape ranging from nearly linear to bell-shaped and have their peaks at values from 2 to 35 cases per 100,000.

In Saint Petersburg (Figure 3A), hepatitis A incidence is predicted to increase continuously, up to 10–30 cases per 100,000 in 2036. The incidence rate in the Sverdlovsk Region (Figure 3D) demonstrates a similar pattern of continuous increase but with significantly lower values, peaking at 3–12 cases per 100,000 in 2036.

With the exception of the Tuva Republic, the predicted dynamics of hepatitis A incidence in the other study regions has a nearly bell-shaped form, with the peak incidence observed in 2028–2032. The peak incidence in Moscow (Figure 3A) is expected to be as high as 8–14 cases per 100,000 in 2031 and 4–10 cases per 100,000 in 2036. The predicted peak incidence in the Republic of Dagestan (Figure 3C) reaches 20–39 cases per 100,000 in 2029, which is followed by a drop back to 0–4 cases. In the Novosibirsk Region (Figure 3E), the peak incidence is around 5–10 cases per 100,000. In Yakutia (Figure 3G), the peak incidence of 12–21 cases per 100,000 is expected in 2031, while in Khabarovsk Region (Figure 3H), hepatitis A incidence peaks at 8–18 cases per 100,000 in 2032 year and slowly decrease thereafter to 5–12 cases per 100,000 in 2036.

In the Tuva Republic (Figure 3F), hepatitis A incidence is projected to be as low as 2 cases per 100,000 in 2024 and then return to a nearly zero incidence.

The pattern of projected national average hepatitis A incidence rate (Figure 3I) is similar to the patterns obtained for the majority of study regions, with a peak at 10–20 cases per 100,000 cases in 2030 and a subsequent gradual decrease to 2–8 cases per 100,000 in 2036.

### 3.4 Estimation of HAV pediatric vaccination threshold

**Figure 4:**
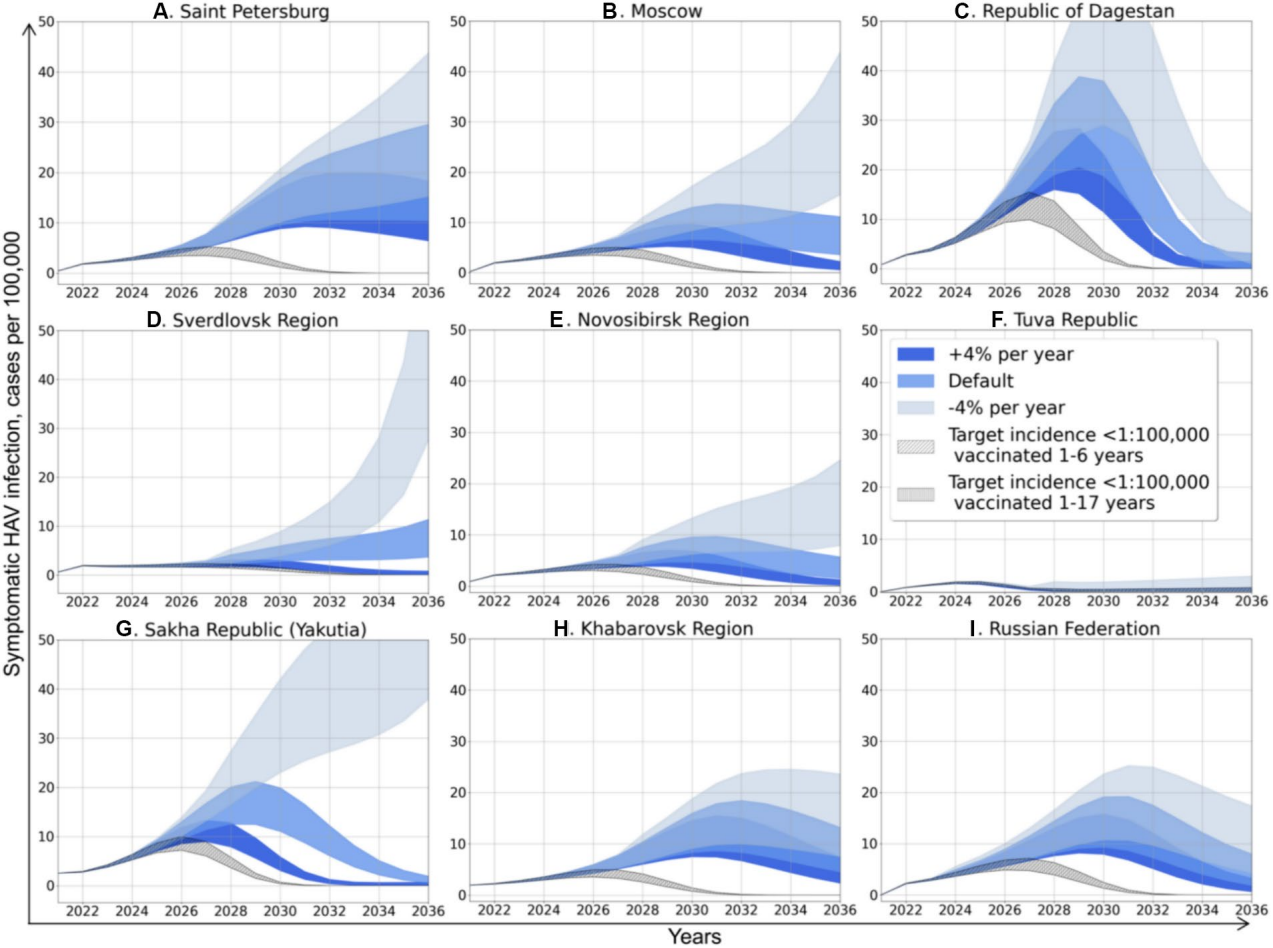
Projected dynamics of symptomatic HAV infection incidence rates according to different scenarios. Medium blue areas depict Scenario 0, light blue areas depict Scenario 1, dark blue areas depict Scenario 2, diagonally hatched areas depict Scenario 3, and vertically hatched areas depict Scenario 4.

The next step of modeling was running five pediatric vaccination scenarios in every study region, as shown in Figure 4.

Scenario 0 assumes that vaccination will remain the same as in 2022. The incidence dynamics related to this scenario is described in detail in the previous subsection and is shown in blue color in Figure 4. Scenario 1 assumes that vaccination will decrease in a geometric progression by 4% each year. It is the most pessimistic scenario, especially for Moscow, the Sverdlovsk Region, the Novosibirsk Region, and Yakutia (Figures 4B,D,E,G), where it will result not only in an incidence surge several times higher compared to Scenario 0, but also a change in the graphs’ shape that becomes close to exponential, suggesting even more significant increase in incidence in the future. In other regions, Scenario 1 does not affect the graphs’ shape, but results in a delayed and higher incidence peak compared to Scenario 0.

Scenario 2 assumes that vaccination will increase geometrically by 4% per year in all regions. This scenario changes the shape of the graphs to bell-shaped, with a 30–50% reduction in the peak incidence compared to Scenario 0.

Scenarios 3 and 4 are hypothetical scenarios required to achieve a symptomatic HAV infection incidence rate in total population below 1 case per 100,000 by 2032. Both scenarios assume an increase in vaccination coverage in terms of arithmetic progression by 2032. Scenario 3 suggests that vaccination coverage is focused on children aged 1 to 6 years old, and Scenario 4 represents the situation of uniform distribution of vaccination in children aged 1 to 17 years. The calculated increase in vaccination rates for both scenarios is shown in Table 4. The required annual increase in HAV vaccination rates varies greatly between regions, from zero in the Tuva Republic to 96–102% in Moscow and amounts to 32% or 35% at the national level, depending on the vaccination scenario. Similarly, the required vaccination coverage by 2032 varies significantly between regions. In Scenario 3, it varies from 40% in the Republic of Dagestan to 100% in the Tuva Republic, with a national average of 69.8%. Scenario 4 requires a lower level of vaccination coverage, although the absolute number of vaccinated persons increases compared to Scenario 3 (Table 4).

**Table 4:** HAV vaccination rates required to achieve an incidence level below 1 case per 100,000 by 2032.

Study region	HAV vaccination in children 1–6 years (Scenario 3)			HAV vaccination in children 1–17 years (Scenario 4)		
	Relative increase per year, %* (95% CI, ±)	Increase per year in absolute number of vaccinated** (95% CI, ±)	Vaccination coverage by 2032, % ***	Relative increase per year, %* (95% CI, ±)	Increase per year in absolute number of vaccinated ** (95% CI, ±)	Vaccination coverage by 2032, %***
Saint Petersburg	42 (4.2)	1,475 (147.5)	68.2	42.5 (4.25)	1,497 (149.7)	30.1
Moscow	96 (10.56)	9,600 (1,056)	60.1	102 (11.22)	10,200 (1,122)	27.6
Republic of Dagestan	20 (1.9)	1,736 (164.92)	40	36 (3.42)	3,125 (3.42)	20.9
Sverdlovsk Region	12 (1.44)	2,718 (326.16)	78	16.8 (2.016)	3,805 (456.6)	43
Novosibrsk Region	18 (1.98)	1,344 (147.84)	69.6	22.2 (7.656)	1,657 (182.27)	35.1
Tuva Republic	0 (0)	0 (0)	100	0 (0)	0	78.1
Sakha Republic (Yakutia)	28 (2.52)	2,372 (213.48)	76.3	32 (2.88)	2,710 (243.9)	55.4
Khabarovsk Region	48 (4.56)	627 (59.565)	75.6	52 (4.94)	680 (64.6)	34.7
Russian Federation, average	32 (3.84)	48,000 (5,760)	69.8	35 (4.2)	52,500 (6,300)	34.8

*Percentage by which the number of vaccinated children should increase annually up until 2032. **The absolute number of vaccinated children by which the number of vaccinated cases should increase annually up until 2032. ***Percentage of vaccinated children by the age of 7 years in Scenario 3 or by the age of 18 years in Scenario 4.

Vaccination Scenarios 3 and 4 result in bell-shaped incidence graphs with the lowest incidence peaks (Figure 4). The incidence graphs for these two scenarios are nearly identical, as the absolute number of susceptible to HAV people who have received the vaccine is similar in both scenarios aiming to achieve the same target incidence rate. However, Scenario 4 requires an unnecessarily larger number of vaccine doses, as more people from the recovered group are vaccinated according to this scenario.

**Figure 5:**
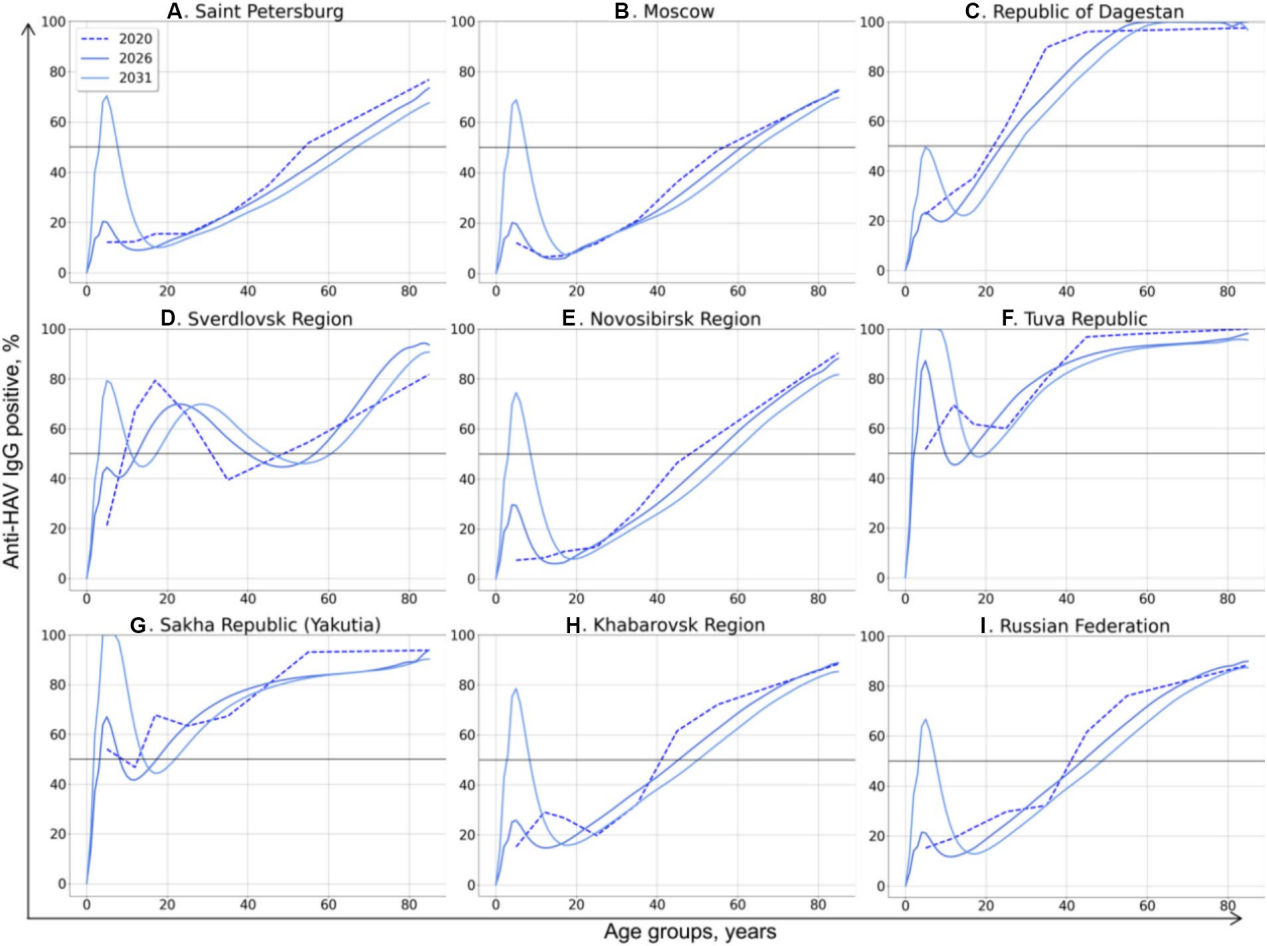
Changes in age-specific HAV seroprevalence rates projected based on Scenario 3: vaccination of children aged 1–6 years with vaccination rates sufficient to achieve an incidence rate below 1 case per 100,000 by 2032. The 50% HAV seroprevalence level is shown as a black solid line.

**Figure 6:**
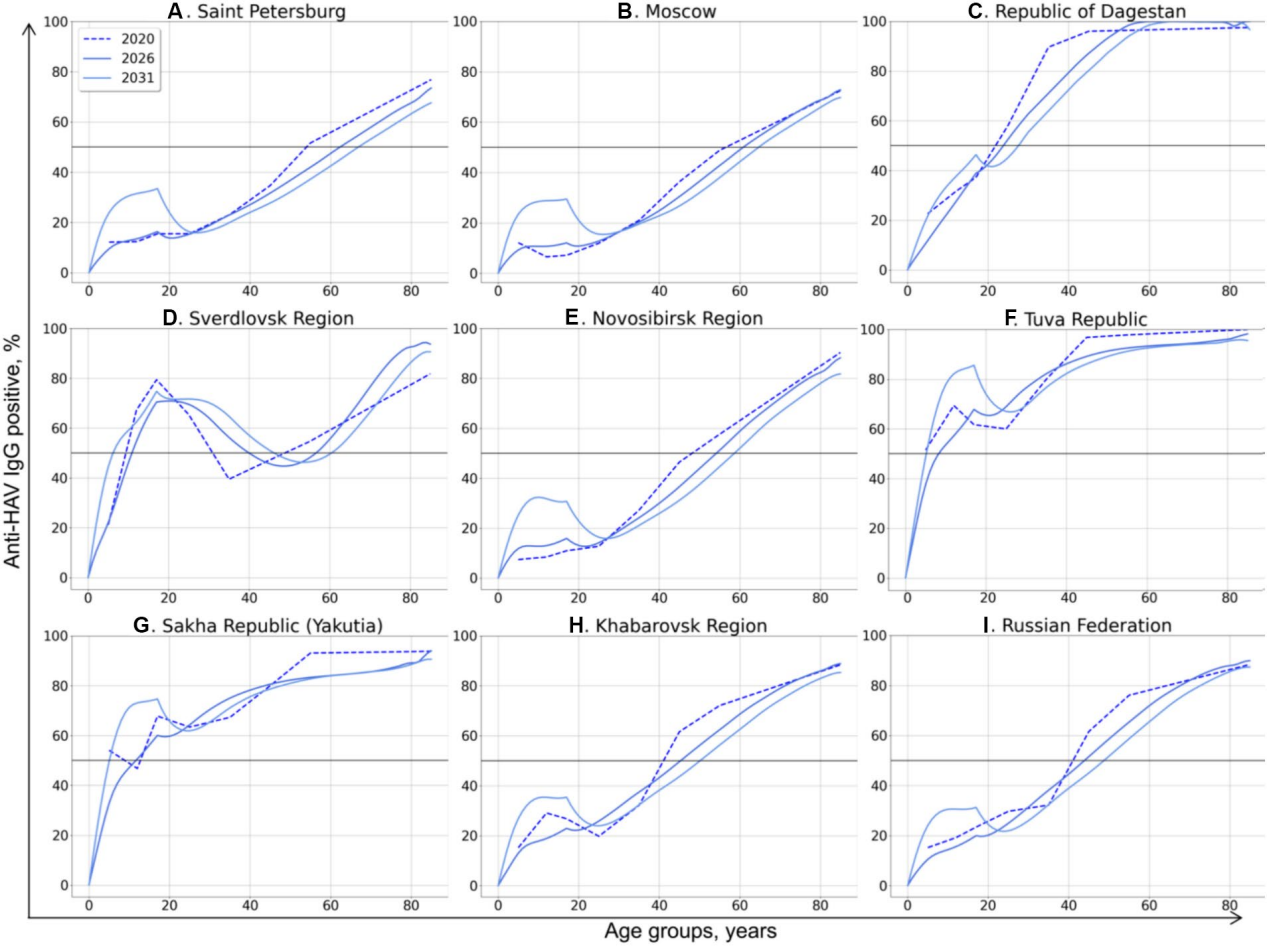
Changes in age-specific HAV seroprevalence rates projected based on Scenario 4: vaccination of children aged 1–17 years with vaccination rates sufficient to achieve an incidence rate below 1 case per 100,000 by 2032. The 50% HAV seroprevalence level is shown as a black solid line.

The SEIRV model runs preformed for Scenario 3 and Scenario 4 allowed us to generate the age-specific HAV seroprevalence rates associated with these scenarios. The predicted changes in HAV seroprevalence rates resulted from Scenario 3 and Scenario 4 are shown in Figures 5, 6, respectively.

In Scenario 3, a drop in the incidence rate below 1 case per 100,000 by 2032 is associated with substantially different HAV seroprevalence levels in children in different regions. In the Republic of Dagestan, a 50% seroprevalence level in the vaccinated generation by 2031 is sufficient to achieve the target incidence rate (Figure 5C). Such a low level of “effective” (i.e., sufficient to prevent almost all symptomatic cases) seroprevalence might be associated with the high incidence rates projected for preceding years in this region (Figure 4C). In contrast, the HAV seroprevalence level in children that is sufficient to achieve the target incidence rate is as high as 100% in the Tuva Republic (Figure 5F) and Yakutia (Figure 5G), probably due to the high transmission rates, as indicated by coefficient β values exceeding 3 for these regions (Table 2). A 70–80% seroprevalence rate in the vaccinated generation is shown to be sufficient to control symptomatic HAV infections in all other regions.

Similar region-specific differences in “effective” HAV seroprevalence in the vaccinated generation are observed in Scenario 4, whereby all children aged 1 to 17 years are vaccinated. In this scenario, a 30% seroprevalence rate is estimated to be sufficient to achieve the target incidence rate below 1 case per 100,000 in Saint-Petersburg, Moscow, the Novosibirsk Region, and the Khabarovsk Region, as well as at the national level (Figures 5A,B,E,H,I). To be effective in the Republic of Dagestan, this vaccination scenario should maintain a HAV seroprevalence rate in children similar to that observed in 2020, reaching 50% by the age of 18 (Figure 6C). Likewise, in the Sverdlovsk Region, vaccination should also maintain the HAV seroprevalence level observed in 2020 (Figure 6D). A seroprevalence level of 85 and 75% in the vaccinated generation is required to achieve control over symptomatic HAV infections in the Tuva Republic (Figure 6F) and Yakutia (Figure 6G), respectively, when Scenario 4 is chosen.

## 4 Discussion

The present study used a compartmental dynamic model to project the shift in herd immunity to HAV and associated changes in disease incidence in the Russian Federation. The main rationale for this study is the hesitancy towards the implementation of a UMV strategy among the country’s healthcare policymakers due to a decline in average national HAV incidence rates in the last two decades, from 57.5 per 100,000 in 2001 to 1.4 per 100,000 in 2022 (17, 18). Thus, the aim of this study was to develop the mathematical model for prediction of hepatitis A epidemiological patterns and, based on results of projections, strengthen the political decision-making regarding the UMV strategy. The age-structured SEIRV compartmental model used in our study presented several differences from mathematical models previously used in other countries that experienced a transition in HAV epidemiology from high to moderate (12, 13). In the present study, the age-structured model took into account the number of vaccinated people in each age group, while immunity induced by inactivated vaccines was considered to be life-long, based on both mathematical prediction data and from real-world observational studies (19–21). In addition, this model used discrete time as this approach is optimal for describing epidemiological dynamics due to the discrete nature of real-world data (22). Also, in contrast to the modeling of HAV epidemiological transition in Thailand (12), Mexico, and Brazil (13), the parameter related to the availability of high-quality drinking water was not included in the analysis since the share of the Russian population provided with high-quality drinking tap water exceeds 85% in recent years (23). Moreover, the majority of HAV cases registered in the country are not due to water-borne infections but are associated with either food-borne infections or person-to-person transmission (17, 24). We did not perform the analysis separately for urban and rural populations, as a previous seroprevalence study demonstrated similar trends in the shift of HAV immunity in both settings (8). However, the calculated values of coefficient β , which defines the average number of people in contact with one infectious person, appeared to be higher in predominantly rural regions compared to urbanized ones, indicating that HAV transmission could still be more intensive in rural settings. A possible limitation of the model presented in this study is the non-inclusion of vulnerable groups in the analysis, such as persons who experience homelessness and men who have sex with men (MSM). These two groups have the highest risk of HAV acquisition in non-endemic countries, with the majority of outbreaks in these groups being associated with person-to-person transmission (25, 26). However, HAV outbreaks have not been registered among homeless people or MSM in the Russian Federation so far, with the exception of isolated imported cases among the latter (27). Moreover, predicting the proportion of people experiencing homelessness using a demographic model is problematic. Thus, the model used in this study assumed that all persons in each age group have the same risk of HAV exposure. However, the implementation of a UMV strategy can eliminate HAV circulation in vulnerable groups in future generations and limit the need for target surveillance and vaccination in these groups, as people would be protected from infection before reaching the age of initiating risk behaviors.

Our model can be used for projection of changes in HAV epidemiological profiles in countries with similar transition from intermediate to low endemicity, where the majority of infections are food-borne or associated with person-to-person transmission. However, this model can be easily supplemented with other variables such as level of sanitation or proportion of people experiencing homelessness, if high quality data available, to meet the country-specific features of HAV epidemiology.

A possible limitation of this study is that the SEIVR model is sensitive to population projections. Thus, the projections presented in our study would only be valid as long as the evolution of the population followed the expected patterns, and no events occurred that would generate sudden changes in the population. In this case, the model should be run with new demographic projections.

The modeling results clearly project a further decrease in HAV herd immunity, with a shift in age at the midpoint of population immunity to older age groups and an increase in incidence, both in the country as a whole and in individual study regions. This projected shift reflects a trend observed in reality in recent decades (8). This trend is especially evident in regions where there is no regional HAV vaccination program. Moreover, the projected bell-shaped incidence curves suggest a possible return of multi-year cyclicality in incidence, which was observed in the Russian Federation up until 2020, with a 5 year average interval between incidence peaks (28). Therefore, the currently observed favorable situation with regard to the incidence of hepatitis A is apparently temporary, and it is projected to be replaced by a rise in incidence above 10–20 symptomatic cases per 100,000 on average if the current level of vaccination is maintained. Moreover, the rate of increase in vaccination in recent years, averaging about 4% per year, does not have a significant impact on the expected incidence levels and is, therefore, insufficient. The projected annual decrease in the rate of vaccination by the same 4% could lead to a devastating increase in symptomatic morbidity and a transition from a bell-shaped incidence curve to a nearly exponential curve, suggesting the possibility of explosive incidence in the future.

Among three regions that have regional mass child vaccination programs (the Tuva Republic, the Sakha Republic (Yakutia), and the Sverdlovsk Region), the projected epidemiological measures for Yakutia are similar to those projected for regions that do not have such a program, indicating the insufficient vaccination coverage in this region. The projected age-specific HAV seroprevalence rates in the Sverdlovsk Region apparently result from a decline in vaccination rates observed in the region in recent years (8). The peak seroprevalence rates that result from the high vaccination coverage rates in children observed in the region before 2014 are shifted to older age groups as these persons grow older. Such a shift in the region is also confirmed by real-world seroprevalence data obtained from 2008 and 2021 serosurveys (8). As can be seen from the projected rates of symptomatic HAV incidence, the decrease in vaccination rates in children that currently occurs in the Sverdlovsk Region might result in a rebound of viral transmission. The Sverdlovsk Region is a clear example of the fact that once a UMV campaign has been started, it cannot be slowed down; otherwise, its effect will be lost. The most obvious possible explanation for the decrease in HAV vaccination rates in the Sverdlovsk Region is the fact that this is a regional vaccination program, i.e., it is financed from the regional budget. As a rule, such regional vaccination programs are financed on a residual basis and can be reduced if there are insufficient funds in the regional budget. This is another argument in favor of including HAV vaccination in the national immunization schedule, which means it is carried out at the expense of the federal budget and, therefore, independent of regional budgets.

The Tuva Republic exhibits a completely different pattern of projected seroprevalence and incidence rates compared to other study regions. In the pre-vaccination era, the Tuva Republic had the highest HAV incidence rates in the country. The single-dose HAV child vaccination in this region started in 2012 and has been accompanied by a high vaccination coverage, which has led to a zero registered incidence since 2016 (29). The results of the mathematical modeling show that, for all scenarios of vaccination coverage at the current level or even at a yearly decrease of 4% relative to the current level, the HAV incidence rate in the Tuva Republic remains very low, both due to the high vaccination rates in children and high seroprevalence levels in adults. However, the estimated vaccination coverage rates required to maintain the incidence rate below one case per 100,000 in the Tuva Republic are the highest among the study regions, indicating the maintenance of transmission risks.

Thus, the results of the HAV epidemic modeling clearly suggest that current vaccination rates, which constitute about 440,000 persons (or 300 per 100,000) per year on average since 2012, are not enough to prevent a future surge of symptomatic infections, showing the advantage of implementing a UMV strategy.

Next, we ran the developed compartmental model to estimate the vaccination threshold required to achieve an incidence rate of less than one case per 100,000 for symptomatic HAV infections following the implementation of a UMV strategy. This level of reported incidence is observed in many non-endemic countries (30) and can be considered as a target level at which hepatitis A is not a public health problem. Two vaccination scenarios with two vaccine doses were simulated. In the first scenario, children aged 1 to 6 years are vaccinated, and in the second scenario, all children from 1 to 17 years are vaccinated. Both scenarios require a substantial yearly increase in vaccination rates to achieve the target incidence level by 2032. However, the scenario of vaccinating children aged 1–6 years appears to be preferable for two reasons. Firstly, this vaccination scenario requires a smaller number of vaccine doses, as the estimated yearly increase in the absolute number of vaccinated children is smaller in this scenario compared to the second scenario. Secondly, the scenario of vaccinating children aged 1–6 years provides a higher estimated vaccination coverage (69.8%) and significantly higher seroprevalence rates (≥70%) in the vaccinated generation. In the scenario of vaccinating children aged 1–17 years, a national average of 34.8% in vaccination coverage is estimated to be sufficient to decrease the incidence of symptomatic HAV infections in the general population. However, such a vaccination coverage rate might be insufficient to prevent outbreaks in high-risk groups in the future among the vaccinated generation. In previous research, the herd immunity and vaccination thresholds were estimated to be 69 and 76%, respectively, among persons experiencing homelessness and persons who use drugs in the United States (14). Similarly, the critical level of herd immunity to combat HAV among MSM to prevent outbreaks was estimated to be 65–70% (31, 32). Thus, the scenario of vaccinating children aged 1–6 years provides a sufficient level of herd immunity that not only controls the HAV incidence in the general population, but also prevents future outbreaks in vulnerable groups in the vaccinated generation.

## 5 Conclusion

The developed compartmental model based on HAV seroprevalence and vaccination data provides insights into a further decline of herd immunity to HAV against the background of ongoing viral transmission. The current favorable situation in regard to hepatitis A morbidity is projected to be replaced by an increase in incidence rates if vaccination coverage remains at the current levels. The obtained results support the introduction of a universal mass vaccination program against hepatitis A in the Russian Federation, with the preferable scenario being when children aged 1 to 6 years are immunized.

## Data availability statement

The original contributions presented in the study are included in the article/Supplementary material, further inquiries can be directed to the corresponding author.

## Ethics statement

The studies involving humans were approved by Independent Interdisciplinary Ethics Committee for the Ethical Review of Clinical Research, Moscow, Russia (Approval No. 17 dated November 16, 2019). The studies were conducted in accordance with the local legislation and institutional requirements. Written informed consent was obtained from all study participants or the participant’s legal guardians/next of kin.

## Author contributions

FT: Investigation, Methodology, Software, Visualization, Writing – original draft. AK: Formal analysis, Validation, Writing – review & editing. KK: Conceptualization, Project administration, Validation, Writing – review & editing. ML: Data curation, Investigation, Writing – review & editing. FK: Data curation, Investigation, Writing – review & editing. VM: Investigation, Resources, Writing – review & editing. VA: Funding acquisition, Resources, Writing – review & editing. MM: Conceptualization, Supervision, Writing – review & editing.

## Footnotes

^1^https://www.graphpad.com/
