# Supplementary material for: Modeling hepatitis A epidemiological profiles and estimating the pediatric vaccination threshold in the Russian Federation
Source: Front Public Health. 2024 Jun 27;12:1371996. doi: 10.3389/fpubh.2024.1371996 (PMC11236541; doi:10.3389/fpubh.2024.1371996)
Supplement: Supplementary file 1 [file Table_1.DOCX]

Supplementary Table S1. Annual hepatitis A incidence rates in the Russian Federation in 1999-2022, cases per 100,000 person

| Saint Petersburg | | | | |
| --- | --- | --- | --- | --- |
| Year | Age groups, years | | | Total population |
|  | 0-14 | 0-17 | ≥18 |  |
| 1999 | 48.23 | No data | 49.41 | 48.00 |
| 2000 | 177.16 | No data | 277.46 | 158.44 |
| 2001 | 159.05 | No data | 235.44 | 145.58 |
| 2002 | 46.51 | No data | 63.76 | 43.63 |
| 2003 | 27.52 | No data | 44.53 | 24.78 |
| 2004 | 109.99 | No data | 172.81 | 100.57 |
| 2005 | 76.92 | No data | 117.61 | 71.11 |
| 2006 | 28.14 | 48.25 | 47.01 | 23.30 |
| 2007 | 14.38 | 21.01 | 20.23 | 9.02 |
| 2008 | 13.80 | 17.76 | 19.88 | 9.22 |
| 2009 | 8.00 | 9.25 | 9.91 | 7.78 |
| 2010 | 6.01 | 6.59 | 5.65 | 5.79 |
| 2011 | 5.06 | 4.70 | 2.51 | 2.82 |
| 2012 | 8.56 | 8.12 | 4.34 | 4.88 |
| 2013 | 8.60 | 8.53 | 4.05 | 4.69 |
| 2014 | 7.19 | 7.19 | 9.20 | 8.91 |
| 2015 | 7.11 | 7.26 | 3.19 | 3.79 |
| 2016 | 10.79 | 9.97 | 4.30 | 5.15 |
| 2017 | 8.06 | 8.45 | 7.14 | 7.34 |
| 2018 | 7.40 | 6.95 | 5.37 | 5.63 |
| 2019 | 5.37 | 4.98 | 3.14 | 3.45 |
| 2020 | 1.20 | 1.19 | 2.10 | 1.94 |
| 2021 | 0.37 | 0.40 | 0.12 | 0.17 |
| 2022 | 2.96 | 5.61 | 2.00 | 2.64 |
| Moscow | | | | |
| Year | Age groups, years | | | Total population |
|  | 0-14 | 0-17 | ≥18 |  |
| 1999 | 38.39 | No data | 40.87 | 37.90 |
| 2000 | 59.59 | No data | 10.37 | 51.31 |
| 2001 | 74.59 | No data | 131.38 | 64.41 |
| 2002 | 41.96 | No data | 63.07 | 38.33 |
| 2003 | 25.45 | No data | 39.43 | 23.15 |
| 2004 | 29.33 | No data | 41.34 | 27.43 |
| 2005 | 27.23 | No data | 38.31 | 25.65 |
| 2006 | 10.48 | 17.22 | 15.49 | 8.87 |
| 2007 | 11.18 | 14.90 | 14.35 | 10.48 |
| 2008 | 5.93 | 9.30 | 8.84 | 5.30 |
| 2009 | 5.59 | 8.23 | 8.22 | 5.12 |
| 2010 | 8.53 | 9.54 | 10.52 | 10.38 |
| 2011 | 5.36 | 5.23 | 2.82 | 3.18 |
| 2012 | 8.36 | 7.84 | 3.72 | 4.34 |
| 2013 | 11.49 | 11.62 | 5.99 | 6.85 |
| 2014 | 7.90 | 7.81 | 11.27 | 10.74 |
| 2015 | 4.94 | 4.52 | 2.07 | 2.46 |
| 2016 | 7.72 | 7.49 | 3.14 | 3.84 |
| 2017 | 6.92 | 7.15 | 9.01 | 8.70 |
| 2018 | 7.87 | 7.78 | 3.74 | 4.43 |
| 2019 | 8.16 | 7.63 | 2.66 | 3.52 |
| 2020 | 1.72 | 1.89 | 1.60 | 1.65 |
| 2021 | 0.54 | 0.47 | 0.14 | 0.20 |
| 2022 | 4.35 | 4.25 | 1.50 | 2.00 |
| Republic of Dagestan | | | | |
| Year | Age groups, years | | | Total population |
|  | 0-14 | 0-17 | ≥18 |  |
| 1999 | 517.77 | No data | 53.72 | 199.3 |
| 2000 | 519.59 | No data | 54.89 | 200.59 |
| 2001 | 262.96 | No data | 38.19 | 107.43 |
| 2002 | 159.62 | No data | 43.48 | 78.53 |
| 2003 | 234.63 | No data | 25.75 | 87.4 |
| 2004 | 199.28 | No data | 25.54 | 75.88 |
| 2005 | 113.64 | No data | 15.66 | 42.92 |
| 2006 | 157.21 | 141.94 | 14.48 | 57.16 |
| 2007 | 201.91 | 173.22 | 9.63 | 62.89 |
| 2008 | 199.19 | 173.52 | 10.83 | 62.31 |
| 2009 | 96.23 | 86.23 | 5.28 | 30.11 |
| 2010 | 36.92 | 35.02 | 3.74 | 12.92 |
| 2011 | 49.45 | 46.78 | 3.17 | 16.53 |
| 2012 | 149.4 | 144.4 | 9.56 | 50.85 |
| 2013 | 104.5 | 98.21 | 7.87 | 35.35 |
| 2014 | 35.55 | 33.85 | 4.49 | 12.56 |
| 2015 | 52.81 | 52.99 | 5.45 | 19.66 |
| 2016 | 25.94 | 23.45 | 3.34 | 9.3 |
| 2017 | 2.96 | 2.94 | 0.57 | 1.27 |
| 2018 | 3.75 | 3.5 | 0.51 | 1.39 |
| 2019 | 3.2 | 3.17 | 0.76 | 1.46 |
| 2020 | 0.13 | 0.34 | 0.41 | 0.39 |
| 2021 | 0 | 0 | 0.05 | 0.03 |
| 2022 | 0.94 | 0.91 | 0.13 | 0.35 |
| Sverdlovsk Region | | | | |
| Year | Age groups, years | | | Total population |
|  | 0-14 | 0-17 | ≥18 |  |
| 1999 | 73.28 | No data | 107.32 | 65.21 |
| 2000 | 50.71 | No data | 97.16 | 40.32 |
| 2001 | 39.94 | No data | 71.76 | 33.21 |
| 2002 | 24.26 | No data | 42.20 | 20.65 |
| 2003 | 48.96 | No data | 100.85 | 39.08 |
| 2004 | 39.98 | No data | 81.71 | 32.42 |
| 2005 | 14.96 | No data | 25.86 | 13.02 |
| 2006 | 8.01 | 8.36 | 6.67 | 7.50 |
| 2007 | 5.31 | 8.36 | 7.83 | 4.58 |
| 2008 | 3.41 | 4.41 | 4.16 | 3.18 |
| 2009 | 2.68 | 5.07 | 5.60 | 2.16 |
| 2010 | 2.77 | 3.04 | 3.72 | 3.6 |
| 2011 | 5.73 | 5.55 | 2.3 | 2.9 |
| 2012 | 10.83 | 10.2 | 5.39 | 6.3 |
| 2013 | 10.45 | 10.45 | 7.2 | 7.8 |
| 2014 | 19.05 | 18.84 | 19.56 | 19.44 |
| 2015 | 11.04 | 10.23 | 8.58 | 8.9 |
| 2016 | 6.15 | 6.16 | 2.64 | 3.3 |
| 2017 | 2.25 | 2.3 | 3.06 | 2.9 |
| 2018 | 4.11 | 4.04 | 2.39 | 2.7 |
| 2019 | 3.37 | 3.03 | 4.06 | 3.84 |
| 2020 | 1.49 | 1.39 | 1.45 | 1.43 |
| 2021 | 0.62 | 0.54 | 0.06 | 0.16 |
| 2022 | 3.85 | 3.41 | 1.28 | 1.74 |
| Novosibirsk Region | | | | |
| Year | Age groups, years | | | Total population |
|  | 0-14 | 0-17 | ≥18 |  |
| 1999 | 15.79 | No data | 10.45 | 17.06 |
| 2000 | 27.13 | No data | 49.21 | 22.17 |
| 2001 | 100.49 | No data | 161.86 | 87.51 |
| 2002 | 26.51 | No data | 49.03 | 22.00 |
| 2003 | 5.40 | No data | 6.67 | 5.16 |
| 2004 | 7.93 | No data | 12.96 | 7.01 |
| 2005 | 25.46 | No data | 48.24 | 21.42 |
| 2006 | 10.36 | 22.94 | 19.43 | 7.05 |
| 2007 | 12.01 | 24.52 | 26.02 | 9.06 |
| 2008 | 5.96 | 12.87 | 13.36 | 4.41 |
| 2009 | 6.52 | 11.76 | 9.42 | 5.36 |
| 2010 | 12.71 | 12.79 | 6.12 | 7.3 |
| 2011 | 12.77 | 14.07 | 7.03 | 8.25 |
| 2012 | 12.25 | 11.69 | 2.86 | 4.39 |
| 2013 | 19.87 | 17.99 | 4.62 | 6.95 |
| 2014 | 20.13 | 19.44 | 8.43 | 10.38 |
| 2015 | 10.36 | 9.32 | 2.16 | 3.46 |
| 2016 | 18.99 | 17.06 | 2.92 | 5.55 |
| 2017 | 15.87 | 15.73 | 7 | 8.68 |
| 2018 | 9.44 | 8.98 | 2.92 | 4.11 |
| 2019 | 13.27 | 12.73 | 4.5 | 6.2 |
| 2020 | 4.58 | 4.12 | 2.39 | 2.75 |
| 2021 | 0.4 | 0.34 | 0 | 0.07 |
| 2022 | 8.5 | 7.65 | 1.4 | 2.72 |
| Tuva Republic | | | | |
| Year | Age groups, years | | | Total population |
|  | 0-14 | 0-17 | ≥18 |  |
| 1999 | 230.16 | No data | 577.46 | 65.15 |
| 2000 | 287.86 | No data | 768.73 | 66.39 |
| 2001 | 284.73 | No data | 803.21 | 55.23 |
| 2002 | 279.64 | No data | 675.85 | 110.63 |
| 2003 | 119.15 | No data | 341.94 | 28.54 |
| 2004 | 134.29 | No data | 401.81 | 29.57 |
| 2005 | 148.99 | No data | 436.59 | 33.01 |
| 2006 | 60.57 | 135.29 | 152.09 | 18.55 |
| 2007 | 45.78 | 116.63 | 134.02 | 7.97 |
| 2008 | 62.46 | 169.75 | 196.91 | 7.35 |
| 2009 | 22.86 | 63.74 | 73.85 | 2.42 |
| 2010 | 458.8 | 400.6 | 8.98 | 137.9 |
| 2011 | 438.9 | 394.9 | 18.19 | 146.1 |
| 2012 | 263.6 | 246.7 | 20.2 | 97.1 |
| 2013 | 6.64 | 7.51 | 1 | 3.2 |
| 2014 | 3.22 | 4.6 | 2.98 | 3.55 |
| 2015 | 1.04 | 1.81 | 0 | 0.6 |
| 2016 | 0 | 0 | 0 | 0 |
| 2017 | 0 | 0 | 0 | 0 |
| 2018 | 0 | 0 | 0 | 0 |
| 2019 | 0 | 0 | 0 | 0 |
| 2020 | 0 | 0 | 0 | 0 |
| 2021 | 0 | 0 | 0 | 0 |
| 2022 | 0 | 0 | 0 | 0 |
| Sakha Republic (Yakutia) | | | | |
| Year | Age groups, years | | | Total population |
|  | 0-14 | 0-17 | ≥18 |  |
| 1999 | 19.77 | No data | 20.35 | 19.55 |
| 2000 | 28.14 | No data | 60.77 | 16.72 |
| 2001 | 61.10 | No data | 104.30 | 46.59 |
| 2002 | 44.15 | No data | 52.88 | 41.32 |
| 2003 | 56.77 | No data | 87.42 | 47.25 |
| 2004 | 56.24 | No data | 123.58 | 36.21 |
| 2005 | 32.64 | No data | 64.08 | 22.85 |
| 2006 | 6.82 | 10.36 | 10.47 | 5.05 |
| 2007 | 6.49 | 4.81 | 5.17 | 7.20 |
| 2008 | 2.31 | 2.62 | 2.85 | 2.19 |
| 2009 | 3.04 | 3.87 | 3.84 | 2.74 |
| 2010 | 7.28 | 6.36 | 2.14 | 3.3 |
| 2011 | 2.4 | 2.37 | 1.56 | 1.8 |
| 2012 | 5.27 | 4.35 | 1.84 | 2.5 |
| 2013 | 2.86 | 2.37 | 2.89 | 0.8 |
| 2014 | 7.53 | 6.7 | 4.56 | 5.13 |
| 2015 | 5.1 | 4.31 | 0.57 | 1.6 |
| 2016 | 1.83 | 1.56 | 0.72 | 0.9 |
| 2017 | 3.59 | 3.08 | 3.72 | 3.6 |
| 2018 | 7.99 | 7.25 | 1.72 | 3.2 |
| 2019 | 1.32 | 1.14 | 1.99 | 1.76 |
| 2020 | 2.22 | 2.27 | 1.28 | 1.6 |
| 2021 | 0.89 | 0.76 | 0 | 0.21 |
| 2022 | 1.78 | 1.51 | 0.42 | 0.72 |
| Khabarovsk Region | | | | |
| Year | Age groups, years | | | Total population |
|  | 0-14 | 0-17 | ≥18 |  |
| 1999 | 57.47 | No data | 123.16 | 40.49 |
| 2000 | 68.87 | No data | 190.53 | 39.34 |
| 2001 | 63.62 | No data | 149.87 | 43.94 |
| 2002 | 86.09 | No data | 166.68 | 68.65 |
| 2003 | 49.40 | No data | 124.87 | 33.98 |
| 2004 | 34.16 | No data | 73.41 | 26.58 |
| 2005 | 36.96 | No data | 92.05 | 26.55 |
| 2006 | 30.31 | 71.71 | 63.93 | 19.04 |
| 2007 | 6.30 | 10.30 | 9.40 | 5.32 |
| 2008 | 2.59 | 2.59 | 3.36 | 2.59 |
| 2009 | 1.40 | 1.14 | 0.98 | 1.46 |
| 2010 | 4.76 | 4.29 | 1.83 | 2.3 |
| 2011 | 9.65 | 9.64 | 2.35 | 3.7 |
| 2012 | 3.56 | 3.77 | 0.63 | 1.2 |
| 2013 | 5.49 | 4.58 | 2.18 | 2.6 |
| 2014 | 6.31 | 7.4 | 7.46 | 7.45 |
| 2015 | 2.83 | 2.43 | 0.46 | 0.8 |
| 2016 | 4.13 | 3.96 | 1.93 | 2.3 |
| 2017 | 4.44 | 3.86 | 2.23 | 2.5 |
| 2018 | 0.86 | 0.75 | 0.37 | 0.45 |
| 2019 | 1.29 | 1.09 | 0.95 | 0.98 |
| 2020 | 1.67 | 1.44 | 0.96 | 1.1 |
| 2021 | 0 | 0 | 1.0 | 1.2 |
| 2022 | 5.85 | 5.75 | 1.26 | 2.22 |
| Russia, average | | | | |
| Year | Age groups, years | | | Total population |
|  | 0-14 | 0-17 | ≥18 |  |
| 1999 | 38.12 | No data | 62.79 | 32.03 |
| 2000 | 56.55 | No data | 120.12 | 41.65 |
| 2001 | 78.95 | No data | 175.24 | 57.48 |
| 2002 | 46.38 | No data | 87.42 | 37.64 |
| 2003 | 2.81 | No data | 62.37 | 21.27 |
| 2004 | 30.19 | No data | 60.12 | 24.45 |
| 2005 | 30.07 | No data | 53.06 | 25.69 |
| 2006 | 15.03 | 29.23 | 28.43 | 10.95 |
| 2007 | 10.27 | 22.39 | 24.08 | 7.25 |
| 2008 | 8.17 | 22.05 | 24.34 | 4.83 |
| 2009 | 7.30 | 7.54 | 19.37 | 4.63 |
| 2010 | 12.13 | 12.39 | 4.93 | 6.3 |
| 2011 | 10.59 | 10.41 | 2.88 | 4.3 |
| 2012 | 14.19 | 13.98 | 3.56 | 5.5 |
| 2013 | 12.71 | 12.66 | 4.22 | 5.8 |
| 2014 | 12.65 | 12.7 | 9.96 | 10.47 |
| 2015 | 9.73 | 9.69 | 3.2 | 4.4 |
| 2016 | 9.18 | 8.9 | 3.32 | 4.4 |
| 2017 | 7.13 | 7.28 | 5.09 | 5.5 |
| 2018 | 4.79 | 4.63 | 2.39 | 2.8 |
| 2019 | 4.93 | 4.78 | 2.39 | 2.88 |
| 2020 | 3.03 | 3 | 1.6 | 1.9 |
| 2021 | 0.4 | 0.37 | 0.06 | 0.12 |
| 2022 | 3.48 | 3.27 | 1.13 | 1.58 |

Supplementary Table S2. Annual HAV vaccination rates in the Russian Federation in 2004-2022

| Saint Petersburg | | | | | | |
| --- | --- | --- | --- | --- | --- | --- |
| Year | Age groups, years | | | | Total population | |
|  | 0-17 |  | Adults (≥18) |  |  |  |
|  | Absolute numbers | per 100,000 | Absolute numbers | per 100,000 | Absolute numbers | per 100,000 |
| 2004 | 15,368 | 1,894.27 | 7,629 | 138.11 | 22,997 | 363.00 |
| 2005 | 11,390 | 1,446.68 | 18,820 | 337.24 | 30,210 | 474.41 |
| 2006 | 7,191 | 946.94 | 26,121 | 463.23 | 33,312 | 520.64 |
| 2007 | 11,542 | 1,160.23 | 8,377 | 153.88 | 19,919 | 309.37 |
| 2008 | 10,426 | 1,089.11 | 9,750 | 177.27 | 20,176 | 312.45 |
| 2009 | 10,194 | 1,096.34 | 6,435 | 115.58 | 16,629 | 255.92 |
| 2010 | 10,134 | 1,102.49 | 5,031 | 89.54 | 15,165 | 231.96 |
| 2011 | 5,159 | 564.70 | 8,392 | 147.12 | 13,551 | 204.76 |
| 2012 | 7,624 | 816.92 | 4,835 | 84.03 | 12,459 | 186.31 |
| 2013 | 6,599 | 695.92 | 5,170 | 88.67 | 11,769 | 173.61 |
| 2014 | 6,447 | 662.57 | 7,687 | 133.46 | 14,134 | 209.93 |
| 2015 | 4,727 | 470.28 | 3,579 | 61.36 | 8,306 | 121.48 |
| 2016 | 4,216 | 403.99 | 4,042 | 68.65 | 8,258 | 119.14 |
| 2017 | 4,029 | 370.14 | 4,416 | 74.88 | 8,445 | 120.89 |
| 2018 | 4,552 | 400.31 | 6,268 | 106.20 | 10,820 | 153.71 |
| 2019 | 5,557 | 453.86 | 6,542 | 109.50 | 12,099 | 168.07 |
| 2020 | 2,973 | 236.46 | 4,966 | 82.83 | 7,939 | 109.46 |
| 2021 | 3,512 | 279.32 | 4,018 | 67.02 | 7,530 | 103.82 |
| 2022 | 3,906 | 304.54 | 5,951 | 99.30 | 9,857 | 135.48 |
| Moscow | | | | | | |
| Year | Age groups, years | | | | Total population | |
|  | 0-17 |  | Adults (≥18) |  |  |  |
|  | Absolute numbers | per 100,000 | Absolute numbers | per 100,000 | Absolute numbers | per 100,000 |
| 2004 | 8,233 | 403.25 | 3,680 | 24.27 | 11,913 | 69.23 |
| 2005 | 10,018 | 473.28 | 14,323 | 93.33 | 24,341 | 139.38 |
| 2006 | 5,590 | 270.64 | 19,060 | 121.85 | 24,650 | 139.21 |
| 2007 | 6,784 | 254.54 | 17,928 | 117.38 | 24,712 | 137.76 |
| 2008 | 23,300 | 891.82 | 27,137 | 175.42 | 50,437 | 278.93 |
| 2009 | 35,336 | 1,371.25 | 11,853 | 75.68 | 47,189 | 258.72 |
| 2010 | 16,353 | 634.26 | 13,513 | 85.37 | 29,866 | 162.26 |
| 2011 | 24,116 | 936.38 | 35,189 | 218.95 | 59,305 | 318.04 |
| 2012 | 21,394 | 768.86 | 29,237 | 182.40 | 50,631 | 269.15 |
| 2013 | 25,817 | 910.19 | 31,138 | 192.31 | 56,955 | 299.33 |
| 2014 | 23,843 | 816.95 | 40,083 | 250.52 | 63,926 | 337.90 |
| 2015 | 23,262 | 772.52 | 44,571 | 276.43 | 67,833 | 354.50 |
| 2016 | 18,744 | 602.63 | 42,692 | 263.13 | 61,436 | 317.74 |
| 2017 | 16,514 | 511.40 | 66,390 | 407.06 | 82,904 | 424.31 |
| 2018 | 22,772 | 679.07 | 89,454 | 546.35 | 112,226 | 568.91 |
| 2019 | 25,892 | 725.94 | 83,231 | 503.04 | 109,123 | 539.88 |
| 2020 | 37,955 | 1,039.54 | 72,565 | 436.07 | 110,520 | 544.65 |
| 2021 | 50,730 | 1,389.43 | 91,132 | 547.64 | 141,862 | 699.11 |
| 2022 | 48,029 | 1,293.33 | 113,438 | 681.20 | 161,467 | 792.82 |
| Republic of Dagestan | | | | | | |
| Year | Age groups, years | | | | Total population | |
|  | 0-17 |  | Adults (≥18) |  |  |  |
|  | Absolute numbers | per 100,000 | Absolute numbers | per 100,000 | Absolute numbers | per 100,000 |
| 2004 | 366 | 57.70 | 0 | 0.00 | 366 | 13.98 |
| 2005 | 90 | 12.47 | 725 | 37.54 | 815 | 30.72 |
| 2006 | 0 | 0.00 | 0 | 0.00 | 0 | 0.00 |
| 2007 | 992 | 115.79 | 9 | 0.48 | 1,001 | 36.59 |
| 2008 | 12,490 | 1,490.57 | 332 | 17.02 | 12,822 | 459.80 |
| 2009 | 13,570 | 1,650.41 | 1,159 | 57.83 | 14,729 | 521.10 |
| 2010 | 2,430 | 300.53 | 32 | 1.55 | 2,462 | 85.82 |
| 2011 | 15,796 | 1,975.64 | 348 | 16.46 | 16,144 | 553.98 |
| 2012 | 22,407 | 2,513.42 | 118 | 5.79 | 22,525 | 768.65 |
| 2013 | 6,892 | 775.91 | 34 | 1.65 | 6,926 | 235.10 |
| 2014 | 15,440 | 1,742.5 | 287 | 14 | 15,727 | 535.44 |
| 2015 | 18,009 | 2,039.16 | 395 | 19.07 | 18,404 | 622.81 |
| 2016 | 46,395 | 5,256.6 | 7,062 | 337.16 | 53,457 | 1,795.58 |
| 2017 | 36,536 | 4,127.3 | 1,466 | 69.22 | 38,002 | 1,265.46 |
| 2018 | 3,346 | 377.25 | 1,160 | 54.16 | 4,506 | 148.77 |
| 2019 | 10,236 | 1157.9 | 3,276 | 149.52 | 13,512 | 439.4 |
| 2020 | 5,557 | 629.8 | 3,172 | 143.13 | 8,729 | 281.72 |
| 2021 | 8,682 | 984 | 8,402 | 379.13 | 17,084 | 551.36 |
| 2022 | 890 | 100.81 | 1,867 | 83.38 | 2,757 | 88.31 |
| Sverdlovsk Region | | | | | | |
| Year | Age groups, years | | | | Total population | |
|  | 0-17 |  | Adults (≥18) |  |  |  |
|  | Absolute numbers | per 100,000 | Absolute numbers | per 100,000 | Absolute numbers | per 100,000 |
| 2004 | 14,068 | 2,023.87 | 10,143 | 271.27 | 24,211 | 546.01 |
| 2005 | 24,883 | 3,677.15 | 12,761 | 343.28 | 37,644 | 856.71 |
| 2006 | 31,164 | 4,830.42 | 13,126 | 353.68 | 44,290 | 1,016.66 |
| 2007 | 50,725 | 5,974.27 | 19,226 | 552.23 | 69,951 | 1,615.28 |
| 2008 | 42,005 | 5,145.61 | 14,808 | 422.63 | 56,813 | 1,315.08 |
| 2009 | 37,527 | 4,753.42 | 14,304 | 405.80 | 51,831 | 1,201.37 |
| 2010 | 24,008 | 3,001.00 | 9,994 | 284.85 | 34,002 | 789.19 |
| 2011 | 25,284 | 3,185.78 | 9,266 | 264.47 | 34,550 | 804.01 |
| 2012 | 27,017 | 3,512.21 | 9,164 | 258.99 | 36,181 | 839.94 |
| 2013 | 46,699 | 5,943.51 | 20,741 | 587.54 | 67,440 | 1,562.62 |
| 2014 | 92,344 | 1,1521.60 | 25,336 | 721.99 | 117,680 | 2,729.95 |
| 2015 | 80,624 | 9,817.65 | 27,252 | 779.29 | 107,876 | 2,498.14 |
| 2016 | 49,017 | 5,806.92 | 22,727 | 653.08 | 71,744 | 1,659.18 |
| 2017 | 37,608 | 4,329.31 | 18,239 | 527.13 | 55,847 | 1,290.15 |
| 2018 | 24,848 | 2,786.00 | 20,066 | 583.69 | 44,914 | 1,037.35 |
| 2019 | 24,487 | 2,646.60 | 17,562 | 517.25 | 42,049 | 973.25 |
| 2020 | 19,042 | 2,038.42 | 15,503 | 458.80 | 34,545 | 800.92 |
| 2021 | 22,648 | 2,424.44 | 13,064 | 386.62 | 35,712 | 827.97 |
| 2022 | 25,661 | 2,735.98 | 14,276 | 424.57 | 39,937 | 928.69 |
| Novosibirsk Region | | | | | | |
| Year | Age groups, years | | | | Total population | |
|  | 0-17 |  | Adults (≥18) |  |  |  |
|  | Absolute numbers | per 100,000 | Absolute numbers | per 100,000 | Absolute numbers | per 100,000 |
| 2004 | 45 | 10.80 | 75 | 3.32 | 120 | 4.49 |
| 2005 | 386 | 95.50 | 49 | 2.17 | 435 | 16.32 |
| 2006 | 377 | 96.40 | 73 | 3.22 | 450 | 16.95 |
| 2007 | 1,124 | 222.30 | 360 | 16.81 | 1484 | 56.06 |
| 2008 | 464 | 94.79 | 297 | 13.79 | 761 | 28.80 |
| 2009 | 113 | 23.73 | 16 | 0.74 | 129 | 4.87 |
| 2010 | 156 | 33.28 | 140 | 6.38 | 296 | 11.12 |
| 2011 | 3,561 | 761.16 | 2,536 | 115.34 | 6,097 | 228.65 |
| 2012 | 1,012 | 219.27 | 577 | 25.93 | 1,589 | 59.14 |
| 2013 | 5,926 | 1,271.40 | 1,848 | 82.38 | 7,774 | 286.92 |
| 2014 | 6,809 | 1,423.30 | 6,874 | 309.77 | 13,683 | 507.25 |
| 2015 | 4,009 | 812.06 | 2,981 | 133.88 | 6,990 | 256.96 |
| 2016 | 14,822 | 2,905.78 | 6,694 | 300.32 | 21,516 | 785.54 |
| 2017 | 5,735 | 1,087.00 | 6,743 | 302.79 | 12,478 | 453.00 |
| 2018 | 8,911 | 1,633.89 | 9,781 | 439.49 | 18,692 | 674.58 |
| 2019 | 11,814 | 2,060.86 | 9,792 | 441.51 | 21,606 | 774.10 |
| 2020 | 8,252 | 1,416.46 | 9,282 | 419.39 | 17,534 | 62.63 |
| 2021 | 7,465 | 1,281.37 | 7,632 | 344.84 | 15,097 | 539.99 |
| 2022 | 8,798 | 1,495.66 | 9,739 | 441.92 | 18,537 | 663.93 |
| Tuva Republic | | | | | | |
| Year | Age groups, years | | | | Total population | |
|  | 0-17 |  | Adults (≥18) |  |  |  |
|  | Absolute numbers | per 100,000 | Absolute numbers | per 100,000 | Absolute numbers | per 100,000 |
| 2004 | 1,029 | 1,177.97 | 0 | 0.00 | 1,029 | 337.42 |
| 2005 | 1,980 | 2,251.18 | 0 | 0.00 | 1,980 | 651.17 |
| 2006 | 138 | 160.22 | 1 | 0.46 | 139 | 45.89 |
| 2007 | 275 | 256.58 | 11 | 5.64 | 286 | 94.59 |
| 2008 | 4,722 | 4,503.06 | 237 | 119.14 | 4,959 | 1,632.42 |
| 2009 | 2,680 | 2,588.32 | 20 | 9.91 | 2,700 | 884.36 |
| 2010 | 5,945 | 5,756.59 | 54 | 26.46 | 5,999 | 1,952.02 |
| 2011 | 18,488 | 17,802.77 | 323 | 158.11 | 18,811 | 6,104.85 |
| 2012 | 41,956 | 40,127.78 | 436 | 212.90 | 42,392 | 13,703.70 |
| 2013 | 12,329 | 11,791.77 | 642 | 311.80 | 12,971 | 4,177.99 |
| 2014 | 14,982 | 13,783.40 | 224 | 111.35 | 15,206 | 4,907.39 |
| 2015 | 8,433 | 7,616.85 | 0 | 0.00 | 8,433 | 2,710.61 |
| 2016 | 6,016 | 5,433.77 | 0 | 0.00 | 6,016 | 1,933.71 |
| 2017 | 10,244 | 9,252.59 | 0 | 0.00 | 10,244 | 3,292.72 |
| 2018 | 8,586 | 7,755.05 | 0 | 0.00 | 8,586 | 2,759.79 |
| 2019 | 7,661 | 6,352.56 | 13 | 10.78 | 7,674 | 2,375.16 |
| 2020 | 6,932 | 5,748.07 | 0 | 0.00 | 6,932 | 2,145.51 |
| 2021 | 6,499 | 5,389.02 | 21 | 17,41 | 6,520 | 2,017.99 |
| 2022 | 8,954 | 7,424.73 | 0 | 0,00 | 8,954 | 2,771.33 |
| Sakha Republic (Yakutia) | | | | | | |
| Year | Age groups, years | | | | Total population | |
|  | 0-17 |  | Adults (≥18) |  |  |  |
|  | Absolute numbers | per 100,000 | Absolute numbers | per 100,000 | Absolute numbers | per 100,000 |
| 2004 | 33,498 | 14,891.38 | 11,982 | 1652.64 | 45,480 | 4,787.52 |
| 2005 | 26,249 | 11,542.13 | 21,813 | 3005.58 | 48,062 | 5,042.33 |
| 2006 | 11,027 | 5,017.29 | 24,792 | 3374.83 | 35,819 | 3,753.06 |
| 2007 | 14,249 | 5,272.14 | 18,508 | 2698.63 | 32,757 | 3,426.11 |
| 2008 | 8,235 | 3,088.12 | 11,121 | 1606.47 | 19,356 | 2,018.50 |
| 2009 | 4,251 | 1,643.72 | 9,036 | 1,291.90 | 13,287 | 1,386.87 |
| 2010 | 4,086 | 1,613.97 | 7,361 | 1,043.86 | 11,447 | 1,194.46 |
| 2011 | 3,490 | 1,396.00 | 6,025 | 850.68 | 9,515 | 992.95 |
| 2012 | 3,635 | 1,435.82 | 7,051 | 1,003.42 | 10,686 | 1,117.95 |
| 2013 | 11,039 | 4,378.80 | 8,014 | 1,139.20 | 19,053 | 1,993.87 |
| 2014 | 21,521 | 8,481.82 | 10,514 | 1,498.93 | 32,035 | 3,353.87 |
| 2015 | 12,210 | 4,788.70 | 8,779 | 1,253.76 | 20,989 | 2,197.36 |
| 2016 | 13,235 | 5,151.33 | 9,715 | 1,389.99 | 22,950 | 2,401.01 |
| 2017 | 11,609 | 4,472.00 | 8,840 | 1,265.21 | 20,449 | 2,133.90 |
| 2018 | 10,331 | 3,942.98 | 8,034 | 1,148.94 | 18,365 | 1,910.51 |
| 2019 | 8,437 | 3,193.50 | 6,319 | 900.81 | 14,756 | 1,528.06 |
| 2020 | 8,418 | 3,187.14 | 4,612 | 653.83 | 13,030 | 1,343.99 |
| 2021 | 8,470 | 3,206.83 | 3,971 | 562.96 | 12,441 | 1,283.23 |
| 2022 | 10,105 | 3,819.79 | 3,888 | 545.73 | 13,993 | 1,432.27 |
| Khabarovsk Region | | | | | | |
| Year | Age groups, years | | | | Total population | |
|  | 0-17 |  | Adults (≥18) |  |  |  |
|  | Absolute numbers | per 100,000 | Absolute numbers | per 100,000 | Absolute numbers | per 100,000 |
| 2004 | 756 | 315.32 | 65 | 5.53 | 821 | 57.97 |
| 2005 | 1,878 | 827.10 | 439 | 37.53 | 2,317 | 165.88 |
| 2006 | 5,632 | 2571.95 | 2,029 | 175.32 | 7,661 | 556.65 |
| 2007 | 1,961 | 696.16 | 718 | 66.59 | 2,679 | 197.00 |
| 2008 | 894 | 330.78 | 191 | 17.61 | 1,085 | 80.08 |
| 2009 | 194 | 73.72 | 14 | 1.29 | 208 | 15.39 |
| 2010 | 397 | 154.83 | 27 | 2.47 | 424 | 31.43 |
| 2011 | 6,607 | 2,576.73 | 248 | 22.83 | 6,855 | 510.47 |
| 2012 | 4,941 | 2,075.22 | 760 | 68.82 | 5,701 | 424.66 |
| 2013 | 38,715 | 16,260.32 | 62,811 | 5689.46 | 101,526 | 7,564.81 |
| 2014 | 7,521 | 3,092 | 395 | 35.94 | 7,916 | 589.74 |
| 2015 | 1,291 | 522.03 | 239 | 21.85 | 1,530 | 114.1 |
| 2016 | 2,931 | 1,161 | 274 | 25.21 | 3,205 | 239.34 |
| 2017 | 2,973 | 1,148.63 | 503 | 46.68 | 3,476 | 260.1 |
| 2018 | 2,099 | 791.3 | 359 | 33.59 | 2,458 | 184.27 |
| 2019 | 1,390 | 501.43 | 1070 | 102.75 | 2,460 | 185.67 |
| 2020 | 3,624 | 1,307.34 | 10,866 | 1043.45 | 14,490 | 1,098.92 |
| 2021 | 1,024 | 369.4 | 663 | 63.67 | 1,687 | 127.94 |
| 2022 | 861 | 309.16 | 257 | 24.95 | 1,118 | 85.45 |
| Russia, average | | | | | | |
| Year | Age groups, years | | | | Total population | |
|  | 0-17 |  | Adults (≥18) |  |  |  |
|  | Absolute numbers | per 100,000 | Absolute numbers | per 100,000 | Absolute numbers | per 100,000 |
| 2004 | 140,991 | 609.34 | 52,982 | 43.72 | 193,973 | 134.39 |
| 2005 | 203,971 | 883.72 | 133,314 | 110.43 | 337,285 | 234.55 |
| 2006 | 240,674 | 1,079.70 | 136,288 | 112.69 | 376,962 | 263.17 |
| 2007 | 294,975 | 1,036.86 | 127,013 | 111.01 | 421,988 | 295.38 |
| 2008 | 346,978 | 1,261.35 | 156,278 | 135.61 | 503,256 | 352.55 |
| 2009 | 208,757 | 782.84 | 93,468 | 80.53 | 302,225 | 211.74 |
| 2010 | 133,338 | 509.42 | 80,260 | 68.80 | 213,598 | 149.54 |
| 2011 | 185,247 | 714.08 | 115,321 | 98.63 | 300,568 | 210.39 |
| 2012 | 238,817 | 911.52 | 150,246 | 128.57 | 389,063 | 271.96 |
| 2013 | 271,800 | 1,030.97 | 237,317 | 202.86 | 509,117 | 355.16 |
| 2014 | 321,459 | 1,203.58 | 198,398 | 170.21 | 519,857 | 362.86 |
| 2015 | 298,003 | 1,081.34 | 177,387 | 151.06 | 475,390 | 327.89 |
| 2016 | 255,710 | 910.95 | 171,491 | 145.31 | 427,201 | 292.42 |
| 2017 | 260,222 | 907.13 | 242,353 | 205.87 | 502,575 | 343.27 |
| 2018 | 162,209 | 553.72 | 307,396 | 261.88 | 469,605 | 320.17 |
| 2019 | 178,081 | 591.67 | 326,035 | 279.30 | 504,116 | 343.33 |
| 2020 | 129,435 | 427.28 | 220,260 | 189.11 | 349,695 | 238.27 |
| 2021 | 181,016 | 597.56 | 255,977 | 219.78 | 436,993 | 297.75 |
| 2022 | 165,932 | 546.24 | 265,351 | 228.59 | 431,283 | 294.47 |
